# Astrocytic Ephrin-B1 Controls Synapse Formation in the Hippocampus During Learning and Memory

**DOI:** 10.3389/fnsyn.2020.00010

**Published:** 2020-03-17

**Authors:** Amanda Q. Nguyen, Jordan Koeppen, Simone Woodruff, Karen Mina, Zoe Figueroa, Iryna M. Ethell

**Affiliations:** ^1^Division of Biomedical Sciences, University of California Riverside School of Medicine, Riverside, CA, United States; ^2^Neuroscience Graduate Program, University of California, Riverside, Riverside, CA, United States; ^3^Cell, Molecular, and Developmental Biology Graduate Program, University of California, Riverside, Riverside, CA, United States

**Keywords:** astrocyte, ephrin-B1, contextual memory, hippocampus, synapse, dendritic spine

## Abstract

Astrocytes play a fundamental role in synapse formation, pruning, and plasticity, which are associated with learning and memory. However, the role of astrocytes in learning and memory is still largely unknown. Our previous study showed that astrocyte-specific ephrin-B1 knock-out (KO) enhanced but ephrin-B1 overexpression (OE) in hippocampal astrocytes impaired contextual memory recall following fear conditioning. The goal of this study was to understand the mechanism by which astrocytic ephrin-B1 influences learning; specifically, learning-induced remodeling of synapses and dendritic spines in CA1 hippocampus using fear-conditioning paradigm. While we found a higher dendritic spine density and clustering on c-Fos-positive (+) neurons activated during contextual memory recall in both wild-type (WT) and KO mice, overall spine density and mEPSC amplitude were increased in CA1 neurons of KO compared to WT. In contrast, ephrin-B1 OE in hippocampal astrocytes impaired dendritic spine formation and clustering, specifically on c-Fos(+) neurons, coinciding with an overall decrease in vGlut1/PSD95 co-localization. Although astrocytic ephrin-B1 influenced learning-induced spine formation, the changes in astrocytic ephrin-B1 levels did not affect spine enlargement as no genotype differences in spine volume were observed between trained WT, KO, and OE groups. Our results suggest that a reduced formation of new spines rather than spine maturation in activated CA1 hippocampal neurons is most likely responsible for impaired contextual learning in OE mice due to abundantly high ephrin-B1 levels in astrocytes. The ability of astrocytic ephrin-B1 to negatively influence new spine formation during learning can potentially regulate new synapse formation at specific dendritic domains and underlie memory encoding.

## Introduction

Hippocampal circuits are known for their plastic nature and play an important role in the formation of new memories and life-long learning (Milner et al., 1998; Neves et al., 2008). Contextual fear learning and retrieval relies on the hippocampus, particularly the CA1 region. This hippocampal-dependent learning requires activation of CA1 pyramidal neurons (Strekalova et al., 2003; Goshen et al., 2011), and promotes the growth and maturation of hippocampal synapses. Indeed, maturation of dendritic spines has been shown to be activity dependent, allowing for the recruitment of AMPARs and an increase in spine volume (Matsuo et al., 2008). In addition to promoting synapse maturation, experience has also been shown to modify hippocampal circuits through selective formation or removal of synapses (Lichtman and Colman, 2000; Draft and Lichtman, 2009; Holtmaat and Svoboda, 2009; Sala and Segal, 2014; Segal, 2017). Therefore, experience and learning can profoundly impact spine turnover rates (Yang et al., 2008; Holtmaat and Svoboda, 2009; Fu et al., 2012; Lai et al., 2012; Sala and Segal, 2014; Segal, 2017). Additionally, learning-induced spine changes are associated with selective spine clustering and formation of “hot spots” on dendrites (Fu et al., 2012; Frank et al., 2018; Lai et al., 2018), which are suggested to allow for efficient storage of information (Hayashi-Takagi et al., 2015; Frank et al., 2018). Most research has focused on neuron–neuron interactions; however, little is known about astrocyte-derived signals that regulate the synaptic remodeling during learning and memory.

Astrocytes play a critical role in maintaining, supporting, and directly modulating neuronal activity and function. Astrocytic processes encapsulate synapses allowing for astrocytes to communicate with neurons. The interactions between astrocytes and synapses can regulate synaptogenesis and pruning, synaptic transmission, and plasticity (Araque et al., 1999; Clarke and Barres, 2013; Chung et al., 2015; Allen and Eroglu, 2017). As these synaptic changes underlie the acquisition, retention, and retrieval of memory, astrocytes are well positioned to influence learning and memory (Nishiyama et al., 2002; Newman et al., 2011; Suzuki et al., 2011; Tadi et al., 2015; Gao et al., 2016; Adamsky et al., 2018). Activation of hippocampal astrocytes was recently suggested to enhance synaptic potentiation and acquisition of contextual fear memory (Adamsky et al., 2018). Astrocytes are also shown to regulate synapse formation, recruitment of AMPARs, and modulating synaptic functions through the release of gliotransmitters, such as glutamate (Fellin et al., 2004), thrombospondin (Christopherson et al., 2005), glypican (Allen et al., 2012), D-serine (Henneberger et al., 2010), and lactate (Alberini et al., 2018). Besides gliotransmission, astrocytes can communicate and affect synaptic functions through contact-mediated factors. Astrocytic contacts with neurons can direct synaptogenesis (Hama et al., 2004; Garrett and Weiner, 2009) and synapse elimination (Chung et al., 2013), which may allow for the refinement of memories.

Trans-synaptic Eph/ephrin-B interactions promote postsynaptic dendritic spine formation and maturation during development (Henderson et al., 2001; Henkemeyer et al., 2003; Kayser et al., 2006) and high levels of EphB receptors and ephrins are retained in the adult hippocampus (Grunwald et al., 2001; Liebl et al., 2003). Furthermore, the loss of EphA4 and EphB2 receptors are reported to affect associative memory in mice (Gerlai et al., 1999; Halladay et al., 2004; Willi et al., 2012; Dines et al., 2015). Interestingly, EphB2 loss affects both short and long-term contextual fear conditioning memory formation, but only long-term memory depends on EphB2 forward signaling (Dines et al., 2015). Disruption of ephrin-B reverse signaling in neurons was also implicated in impaired hippocampal-dependent learning and memory in EphB2 KO mice (Grunwald et al., 2001). In addition, ephrin-B2 expression is upregulated in CA1 neurons but not the cortex or amygdala following fear conditioning without changes in levels of EphA4 receptor (Trabalza et al., 2012). While ephrin-B2 can activate both EphA4 and EphB receptors, ephrin-B1 is known for its high affinity for EphB but not EphA4 receptors. Deletion of neuronal ephrin-B1 was also responsible for impaired contextual recall in ephrin-B1 KO mice following fear conditioning (Arvanitis et al., 2014). Mutations in the *efnb1 gene* that encodes ephrin-B1 are associated with CranioFrontalNasal Syndrome, characterized by hypertelorism, frontonasal dysplasia, coronal craniosynostosis, and mild learning disability (Twigg et al., 2004; Wieland et al., 2004). However, little is known about the role of astrocytic ephrin-B1. We previously reported that deletion and overexpression (OE) of astrocytic ephrin-B1 in the adult CA1 hippocampus affects contextual memory (Koeppen et al., 2018), but the mechanism is still not clear.

Our new findings suggest that astrocytic ephrin-B1 influences hippocampal-dependent contextual memory by regulating new dendritic spine formation and clustering on hippocampal neurons activated during memory recall, without affecting spine maturation. While we found that both wild-type (WT) and astrocytic ephrin-B1 knock-out (KO) mice showed a significant increase in dendritic spine density and clustering on activated c-Fos(+) neurons compared to c-Fos(-) neurons following contextual recall, dendritic spine density remained higher in trained KO compared to WT, which coincided with a greater vGlut1/PSD95 co-localization and enhanced excitatory postsynaptic currents (EPSCs) in CA1 neurons of KO mice. In contrast, astrocytic ephrin-B1 overexpressing (OE) mice showed no increase in dendritic spine density and clustering on c-Fos(+) neurons compared to c-Fos(-) neurons, which coincided with an overall decrease in vGlut1/PSD95 co-localization. However, changes of ephrin-B1 levels in astrocytes did not affect spine enlargement, as no genotype differences in spine volume were observed between trained WT, KO, and OE groups. Our results suggest that the deficits in dendritic spine formation and clustering, but not spine maturation, may underlie impaired contextual memory recall in OE mice. These studies implicate astrocytic ephrin-B1 as a negative regulator of synapse formation in the activated hippocampal neurons during learning, which can influence contextual memory. Future studies will determine whether activity-dependent up-regulation or down-regulation of ephrin-B1 levels in selective astrocytes controls addition or removal of synapses on specific neurons or dendrites, which may potentially underlie memory encoding.

## Materials and Methods

### Mice

All animal care protocols and procedures were approved by the UC Riverside Animal Care & Use Program and done according to NIH and Institutional Animal Care and Use Committee guidelines; animal welfare assurance number A3439-01 is on file with the Office of Laboratory Animal Welfare (OLAW). Mice were maintained in an AAALAC accredited facility under 12-h light/dark cycle and fed standard mouse chow. ERT2-Cre*^*GFAP*^* male mice (B6.Cg-Tg(*GFAP*-cre/ERT2)505Fmv/J, RRID: IMSR_JAX:012849) were crossed with *ephrin-B1*^flox/ +^ female mice (129S-*Efnb1*^tm 1Sor^/J, RRID: IMSR_JAX:007664) to obtain ERT2-Cre*^*GFAP*^ephrin-B1*^*flox/y*^ (KO) or ERT2-Cre*^*GFAP*^ephrin-B1*^+/y^ (WT) male mice. Postnatal day (P) 70–90 adult WT and KO littermates received intraperitoneal (IP) injection of tamoxifen (TAM) (1 mg in 5 mg/ml of 1:9 ethanol/sunflower seed oil solution) once a day for 7 consecutive days. There were no detectable changes in ephrin-B1 levels in astrocytes or neurons of TAM-injected WT mice (not shown). In TAM-treated KO mice, ephrin-B1 immunoreactivity was observed only in neuronal cell bodies and dendrites of the CA1 hippocampus, but was significantly reduced in hippocampal astrocytes as previously reported (Nikolakopoulou et al., 2016; Koeppen et al., 2018). Genotypes were confirmed by PCR analysis of genomic DNA isolated from mouse tails.

### Stereotaxic Microinjections

Expression of ephrin-B1 and tdTomato was induced in hippocampal astrocytes via adeno-associated viruses (AAV7) containing *AAV7.GfaABC1D.ephrin-B1.SV40* [AAV-ephrin-B1; viral titer at 7.56 × 10^12^ viral particles (VP)/ml] or *AAV7.GfaABC1D.tdTomato.SV40* (AAV-tdTomato; viral titer at 4.46 × 10^12^ VP/ml), respectively (both obtained from UPenn Vector Core^1^). VP were concentrated with Amicon ultra-0.5 centrifugal filter (UFC505024, Sigma-Aldrich), which was pretreated with 0.1% Pluronic F-68 non-ionic surfactant (24040032, Thermo Fisher). Mice were anesthetized with IP injections of ketamine/xylazine mix (80 mg/kg ketamine and 10 mg/kg xylazine). To ensure for adequate anesthesia, paw pad pinch test, respiratory rhythm, righting reflex, and/or loss of corneal reflex were assessed. Adult P70-90 Thy1-EGFP mice (RRID: IMSR_JAX: 007788) received craniotomies (1 mm in diameter) and VPs were stereotaxic injected into the dorsal hippocampus (2.5 mm posterior to bregma, 1.0 mm lateral to midline, and 1.2 mm from the pial surface). Control mice were bilaterally injected with 2 μl of 1.16 × 10^13^ VP/ml AAV-tdTomato, and experimental animals received bilateral injection of 1 μl of 3.78 × 10^13^ VP/ml AAV-ephrin-B1 + 1 μl of 2.32 × 10^13^ VP/ml AAV-tdTomato. Post-surgery, mice received 0.3 ml of buprenorphine by subcutaneous injection every 8 h for 48 h, as needed for pain. Animals were allowed to recover for 14 days prior to fear conditioning tests and/or immunohistochemistry. There was a significant four-fold increase in ephrin-B1 immunoreactivity in CA1 hippocampal astrocytes of mice injected with AAV-ephrin-B1 + tdTomato (OE) compared to AAV-tdTomato (WT) as previously reported (Koeppen et al., 2018). Mice showing bilateral hippocampal tdTomato expression were used for the analysis.

### Fear Conditioning Test

A fear-conditioning paradigm was used to assess hippocampal dependent contextual learning as previously described (Anagnostaras et al., 2001; Koeppen et al., 2018). Two contexts were used to test contextual memory. Context A was an 18 × 18 cm rectangular clear plexiglass box with 16-grated steel bar flooring; trials in context A were in white light and the scent of Quatricide TB. Context B was in a cylinder with a diameter of 15 cm and a height of 20 cm and checkered black and white walls; trials in context B were in altered light with fresh litter and the scent of Windex. Animals were allowed to acclimate in the behavioral room for 30 min before each testing day and handled for 2 min for 5 days prior to testing. On day 1, the test mouse was placed in context A and habituated to the chamber for 10 min, 1 h after context A mice were habituated to context B for 10 min. The mouse was removed and separated from its home cage until all mice in that cage were habituated to both contexts. On day 2, test mice were trained to associate an unconditioned stimulus (US; 0.6 mA scrambled foot shock) with a conditioned stimulus (CS; 9 kHz, 70 dB tone) in context A. Initially, test mice were placed in context A and given 3 min for habituation, then followed by a 30 s tone (CS), which co-terminated with a 2 s foot shock (US). The CS–US pairing occurred five times, with a pseudorandom interval between pairings. The test mouse, again, was removed and separated from its home cage until all mice in that cage were trained. On day 3, animals were tested for their associated memory of the context (in context A) and of the CS tone (in context B). For contextual recall, mice were placed in context A for 5 min with no sound and returned to home cage for 1 h before testing context B. For tone recall test, mice were placed in context B for a total of 6 min, with the CS tone playing for the final 3 min. Control mice were taken directly from their home cage in the vivarium and immediately perfused and did not undergo the fear conditioning paradigm. For dendritic spine analysis and immunohistochemistry, three to four animals were euthanized and perfused 1 h after context A contextual recall only. Animals undergoing both context A and context B recall were euthanized and perfused 1 h after context B tone recall. Freezing behavior was measured as a percentage of time freezing using TopScan Software. GraphPad Prism 6 software (RRID: SCR_002798) was used to perform a one-way ANOVA followed by Tukey’s *post hoc* analysis or *t*-test when appropriate, data represent mean ± SEM.

### Immunohistochemistry

Animals were anesthetized with isoflurane and transcardially perfused first with 0.9% NaCl, followed by fixation with 4% paraformaldehyde (PFA) in 0.1 M phosphate-buffered saline (PBS), pH 7.4. Brains were post-fixed overnight with 4% PFA in 0.1 M PBS and sectioned into 100 μm coronal slices with a vibratome. Excitatory presynaptic boutons were labeled by immunostaining against vesicular glutamate transporter 1 (vGlut1) using rabbit anti-vGlut1 antibody (0.25 mg/ml, Invitrogen Cat# 482400, RRID: AB_2533843), postsynaptic sites were identified with mouse anti-postsynaptic density-95 (PSD95) antibody (1.65 μg/ml, Invitrogen Cat# MA1-045, RRID: AB_325399). Inhibitory sites were detected with mouse anti-glutamic acid decarboxylase 65 (GAD65) antibody (10 μg/ml, BD Pharmingen Cat# 559931, RRID: AB_397380). Parvalbumin (PV)-positive cells were identified with mouse anti-PV antibody (6 μg/ml, Sigma-Aldrich Cat# P3088, RRID: AB 477329). Activated neurons were detected with anti-c-Fos antibodies (40 μg/ml, Invitrogen Cat# PA1-37437, RRID: AB_1073599). The secondary antibodies used were Alexa Fluor 594-conjugated donkey anti-mouse IgG (4 mg/ml, Molecular Probes Cat# A-21203, RRID: AB_141633), Alexa Fluor 647-conjugated donkey anti-rabbit IgG (4 mg/ml, Molecular Probes Cat# A-31573, RRID: AB_2536183), Alexa Fluor 647-conjugated donkey anti-goat IgG (4 mg/ml, Molecular Probes Cat# A-21447, RRID: AB_141844), or Alexa Fluor 488-conjugated donkey anti-goat IgG (4 mg/ml, Molecular Probes Cat# A-11055, RRID: AB_2534102). Sections were mounted on slides with Vectashield mounting medium containing DAPI (Vector Laboratories Inc. Cat# H-1200, RRID: AB_2336790).

### Confocal Imaging and Analysis

Confocal images of the stratum radiatum (SR) and stratum lacunosum-moleculare (SLM) layers of dorsal CA1 hippocampus were taken with a Leica SP2 and LSM 880 Airyscan Carl Zeiss confocal laser-scanning microscope. A series of high-resolution optical sections (1,024 × 1,024-pixel format) were captured with a 20× or 63× water-immersion objective (1.2 numerical aperture) and 1× zoom at 1-μm step intervals (*z*-stack of 10 optical sections). All images were acquired under identical conditions. For the analysis of vGlut1, GAD65, PSD95, and PV immunolabeling, at least six sequential images were captured for a selected area at 1-μm step intervals; each image in the series was threshold-adjusted to identical levels (0–160 intensity), and puncta (0.5–10 μm^2^) were measured using ImageJ software (RRID: nif-0000-30467). Three adjacent areas from SR and SLM were imaged and analyzed per each hippocampus from at least three animals/group. Colocalization of vGlut1/PSD95 and vGlut1/PV was analyzed using ImageJ plugin for colocalization.^2^ Statistical analysis was performed with one-way ANOVA followed by Tukey’s *post hoc* analysis or *t*-test when appropriate using GraphPad Prism 6 software (RRID: SCR_002798), data represent mean ± standard error of the mean (SEM).

### Dendritic Spine Analysis

Dendritic spines were analyzed in dorsal CA1 hippocampus with GFP using transgenic Thy1-GFP-M mice [Tg(Thy1-EGFP)MJrs/J, RRID: IMSR_JAX:007788] for ephrin-B1 OE condition and Diolistic approach (Henkemeyer et al., 2003) in ephrin-B1 KO mice. Animals were anesthetized with isoflurane and transcardially perfused initially with 0.9% NaCl, followed by fixation with 4% PFA in 0.1 M PBS, pH 7.4. Brains were post-fixed for 2 h in 4% PFA in 0.1 M PBS, and 100 μm coronal sections were cut with a vibratome. Dendritic spines were labeled in ephrin-B1 KO mice and their WT counterparts using a DiOlistic approach (Henkemeyer et al., 2003) using fluorescent lipophilic dye 1,1′-dioctadecyl-3,3,3′,3′-tetramethyl-indocarbocyanine perchlorate (DiO, D3898, Molecular Probes) coating tungsten particles. DiO was delivered by helium-powered ejection (Bio-Rad Helios Gene Gun System) into hippocampal slices and incubated in 0.1 M PBS for 72 h. CA1 hippocampal neurons were imaged using LSM 880 Airyscan Carl Zeiss confocal microscope. Ten to fifteen DiO-labeled or GFP-expressing neurons were randomly selected per group, and dendrites were imaged using a 63× objective (1.2 NA), 1× zoom. Three-dimensional fluorescent images were created by the projection of each *z*-stack containing 50–100 high-resolution optical serial sections (1,024 × 1,024-pixel format) taken at 0.5 μm intervals in the *X*–*Y* plane. Quantifications of the spine density (spines per 10 μm dendrite), lengths (μm), volumes (μm^3^), and inter-spine intervals (μm) were carried out using Neurolucida 360 software (MicroBrightField RRID: SCR_001775). We observed an overall higher density of spines in DiO-labeled WT neurons compared to GFP-expressing WT neurons, which was most likely due to a better detection of smaller spines with membrane dye DiO than GFP. There were about 60–70% of smaller spines in DiO labeled WT neurons compared to 50–55% of smaller spines in GFP-expressing WT neurons (Table 1). Therefore, comparisons were made only between DiO-expressing WT and KO groups or GFP-expressing WT and OE groups. Statistical analysis was performed with two-way ANOVA followed by Bonferroni’s *post hoc* analysis using GraphPad Prism 6 software (GraphPad Prism, RRID: SCR_002798), data represent mean ± SEM.

**TABLE 1:** (Extended data table supporting Figures 1C–E.) Average dendritic spine density, length and volume in Fos(−) and c-Fos(+) CA1 neurons of WT and KO mice.

			Spine distribution (%)		
	Spine density (spines/10 μm)	Spine length (μm)	0–0.5 μm^3^	0.5–1.0 μm^3^	>1.0 μm^3^
**WT**					
*c-Fos*(−) (*n* = 10)	10.42 ± 0.68	2.31 ± 0.20	69.35 ± 1.86	24.73 ± 2.12	5.92 ± 0.58
*c-Fos*(+) (*n* = 12)	13.27 ± 0.57*	2.74 ± 0.08*	59.62 ± 3.49*	29.23 ± 2.20	11.15 ± 1.80*
**KO**					
*c-Fos*(−) (*n* = 11)	12.37 ± 0.99	2.36 ± 0.03	71.06 ± 2.32	24.12 ± 1.86	4.80 ± 0.78
*c-Fos*(+) (*n* = 11)	15.98 ± 0.78**	2.47 ± 0.04	60.03 ± 2.04**	29.87 ± 1.17	10.15 ± 1.42*

Statistical analysis was performed using two-way ANOVA (genotype and c-Fos as factors) with Bonferroni’s post hoc test; c-Fos(−) versus c-Fos(+): *P < 0.05, **P < 0.01.

### Synaptosome Purification

Synaptosome purification was performed as previously described (Hollingsworth et al., 1985). Briefly, hippocampal tissues were homogenized in 1 ml synaptosome buffer (124 mM NaCl, 3.2 mM KCl, 1.06 mM KH_2_PO_4_, 26 mM NaHCO_3_, 1.3 mM MgCl_2_, 2.5 mM CaCl_2_, 10 mM Glucose, 20 mM HEPES), then filtered through a 100 μm nylon net filter (NY1H02500, Millipore) and 5 μm nylon syringe filter (SF15156, Tisch International). Homogenate flow through was collected, and synaptosomes were spun down at 10,000 × *g*, at 4°C, for 30 min. Synaptosomes were resuspended in 800 μl synaptosome buffer and processed for western blot analysis.

### Western Blot Analysis

Tissue homogenate or purified synaptosome samples were centrifuged at 10,000 × *g*, 4°C, for 30 min. Pellets were re-suspended in lysis buffer (50 mM Tris, 100 mM NaCl, 2% TritonX-100, 10 mM EDTA) containing 2% protease inhibitor cocktail (P8340, Sigma-Aldrich) and incubated for 2 h at 4°C. Samples were added to 2× Laemmli Buffer (S3401, Sigma-Aldrich) and run on an 8–16% Tris-Glycine Gel (EC6045BOX, Invitrogen). Protein samples were transferred onto a nitrocellulose blotting membrane (10600007, GE Healthcare). Blots were blocked with 5% milk in TBS (10 mM Tris, 150 mM NaCl, pH 8.0), followed by immunostaining with mouse anti-PSD95 (1.65 μg/ml, Invitrogen Cat# MA1-045, RRID: AB_325399), rabbit anti-GluA1 (1:100, Millipore Cat# AB1504, RRID: AB_2113602), rabbit anti-GluA2/3 (0.1 μg/ml, Millipore Cat# AB1506, RRID: AB_90710), or mouse anti-GAPDH (0.2 μg/ml, Thermo Fisher Scientific Cat# 39-8600, RRID: AB_2533438) antibodies in 0.1% tween 20/TBS at 4°C for 16 h. The secondary antibodies used were HRP conjugated donkey anti-mouse IgG (Jackson ImmunoResearch Cat#715-035-150, RRID: AB_2340770) or HRP conjugated goat anti-rabbit IgG (Jackson ImmunoResearch Cat# 111-035-003, RRID: AB_2313567). Blots were incubated in ECL 2 Western Blotting Substrate (Pierce Cat# 80196) and a signal was collected with CL-XPosure film (34090, Pierce). Band density was analyzed by measuring band and background intensity using Adobe Photoshop CS5.1 software (RRID: SCR_014199). Statistical analysis was performed with a one-way ANOVA followed by Tukey’s *post hoc* analysis or *t*-test when appropriate using GraphPad Prism 6 software (RRID: SCR_002798), data represent mean ± SEM.

### Electrophysiology

Brain slices were obtained from naïve or trained adult mice (P90-110) 1 h after recall test. Animals were deeply anesthetized with isoflurane and decapitated. Mouse brains were rapidly removed and immersed in ice-cold “slushy buffer” with high Mg^2+^ and sucrose concentration containing the following (in mM): 87 NaCl, 75 sucrose, 2.5 KCl, 0.5 CaCl_2_, 7 MgCl_2_, 1.25 NaH_2_PO_4_, 25 NaHCO_3_, 10 glucose, 1.3 ascorbic, acid, 0.1 kynurenic acid, 2.0 pyruvate, and 3.5 MOPS with a pH of 7.4 and saturated with 95% O_2_/5% CO_2_. Transverse hippocampal slices (350 μm) were prepared by using a vibrating blade microtome (Campden 5100mz-Plus, Campden Instruments Ltd.) and transferred into a holding chamber containing oxygenated ACSF (in mM; 125 NaCl, 2.5 KCl, 2.5 CaCl_2_, 1.3 MgCl_2_, 1.25 NaH_2_PO_4_, 26 NaHCO_3_, 15 glucose, 3.5 MOPS with a pH of 7.4) for 1 h at 33°C. Slices were then transferred to a submersion recording chamber continually perfused with oxygenated ACSF at a flow rate of 1 ml/min. Slices were allowed to equilibrate for approximately 10 min to reach a stable baseline response prior to running experimental protocols.

Blind whole-cell patch experiments were performed as described (Castaneda-Castellanos et al., 2006). Tight-seal whole-cell voltage clamp recordings were obtained using pipettes made from borosilicate glass capillaries pulled on a Narishige PC-10 vertical micropipette puller (Narishige, Tokyo, Japan). Pipette resistance ranged from 3 to 4 MΩ, filled with an internal solution containing (in mM) 130 CsOH, 130 D-gluconic acid, 0.2 EGTA, 2 MgCl_2_, 6 CsCl, 10 HEPES, 2.5 ATP-Na, 0.5 GTP-Na, 10 phosphocreatine, and 0.1% biocytin for cellular post labeling, pH adjusted to 7.2–7.3 with CsOH, osmolarity adjusted to 300–305 mOsm with ATP-Na. The series resistance was <25 MΩ and was compensated, if the series resistance changed >20% during the course of an experiment, the data were discarded. For evoked EPSCs and IPSCs, electrical stimuli (0.1 Hz) were delivered through a bipolar, Teflon^®^-coated tungsten electrode placed in the SR region and close proximity to the recording electrode. Neurons were voltage-clamped at either −70 mV to measure AMPAR evoked responses or +40 mV to measure NMDA receptor evoked responses. All EPSCs were recorded in the presence of 50 μM picrotoxin, a GABA_A_ receptor antagonist, to block GABA_A_-mediated currents at 33°C. To measure inhibitory postsynaptic currents (IPSCs), neurons were voltage-clamped at 0 mV with 10 μM NBQX, an AMPA receptor antagonist, and 50 μM D-AP5, a NMDA receptor antagonist at 33°C. 1 μM tetrodotoxin was added to isolate mEPSC and mIPSC responses. EPSCs and IPSCs were recorded using an EPC-9 amplifier (HEKA Elektronik, Lambrecht, Germany), filtered at 1 kHz, digitized at 10 kHz, and stored on a personal computer using pClamp 10.7 software (Molecular Device) to run analysis. AMPA, NMDA-mediated EPSCs, IPSCs evoked responses, mEPSCs, and mIPSCs were analyzed by Clampfit 10.7 software (Molecular Device). All averaged data were presented as means ± SEM. Statistical significance was determined by Student’s *t*-test using Prism 7.0 software (GraphPad Software, Avenida, CA, United States).

## Results

We previously reported that the loss of astrocytic ephrin-B1 in adult mice resulted in enhanced contextual recall, while OE of ephrin-B1 in the adult hippocampal astrocytes impaired contextual memory recall (Koeppen et al., 2018). The goal of this study was to understand the mechanism by which astrocytic ephrin-B1 affects contextual fear conditioning memory, in particular how the deletion or OE of astrocytic ephrin-B1 affects remodeling of synapses and dendritic spines in the CA1 hippocampus following contextual learning. To accomplish this, astrocyte-specific ephrin-B1 KO and ephrin-B1 OE mice, with corresponding WT counterparts, were trained in a fear condition paradigm to associate a context with an electric shock. Next day, the mice were placed in context A, in which they were trained, and their freezing was evaluated as a measure of contextual memory (Supplementary Figure 1). Dendritic spine density, morphology, and clustering were analyzed in the CA1 hippocampus of these mice 1 h after contextual memory recall. As specific memories are encoded in a sub-set of hippocampal neurons (Liu et al., 2012; Tonegawa et al., 2015), we further analyzed dendritic spine changes in CA1 hippocampal pyramidal neurons that were activated (c-Fos+) or not activated (c-Fos−) during contextual memory recall. Additionally, changes in the excitatory synaptic sites were analyzed by co-labeling of vGlut1 with PSD-95 puncta in CA1 hippocampus.

### Dendritic Spine Density Is Higher on CA1 Hippocampal Neurons of Ephrin-B1 KO Mice, Specifically on cFos(+) Neurons That Are Activated During Contextual Recall

**FIGURE 1:**
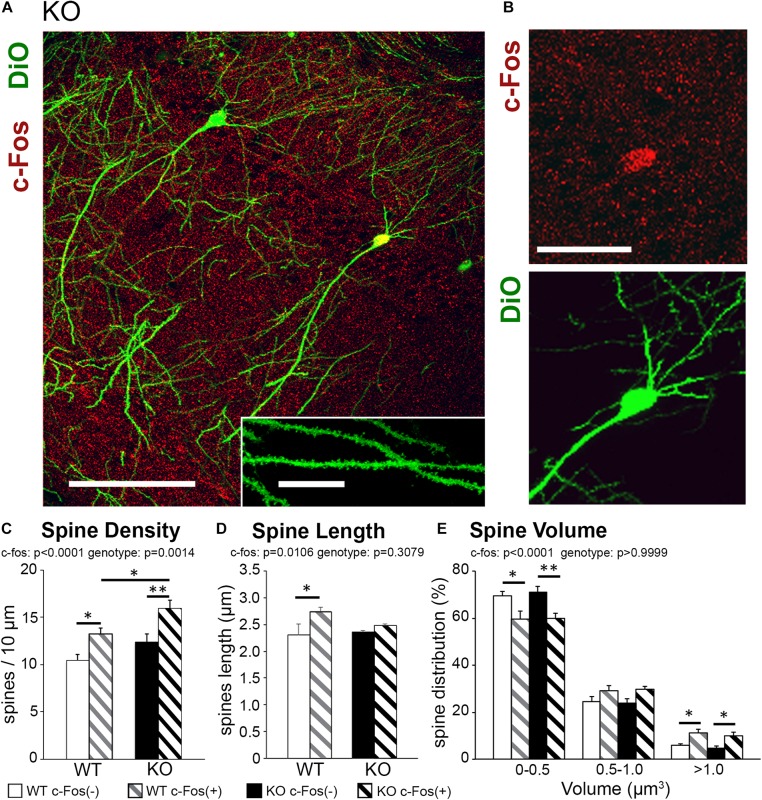
Learning-induced spine formation is observed on CA1 neurons in astrocyte-specific ephrin-B1 KO mice, specifically on cFos(+) neurons that are activated during contextual recall. **(A)** Confocal image showing DiO (green) and c-Fos (red) labeled neurons in CA1 hippocampus of adult KO mice, scale bar is 150 μm. High magnification shows examples of dendritic spines, scale bar is 20 μm (insert). **(B)** High magnification image of CA1 pyramidal neuron showing c-Fos(+) immunoreactivity (red) and DiO labeling (green). **(C–E)** Graphs show the average number of dendritic spines per 10 μm dendrite **(C)**, spine length **(D)**, and spine volume **(E)** in c-Fos(+) and c-Fos(–) CA1 neurons from WT and KO mice. **(C)** There is a significant increase in average dendritic spine density in c-Fos(+) neurons compared to c-Fos(–) neurons in KO mice [two-way ANOVA, c-Fos *F*(1,48) = 19.91, *p* < 0.0001; genotype *F*(1,48) = 11.55, *p* = 0.0014; Bonferroni’s *post hoc* test, ^∗∗^*p* < 0.0066 c-Fos(+) KO vs. c-Fos(–) KO; ^∗^*p* = 0.0422 c-Fos(+) WT vs. c-Fos(–) WT]. We also observed higher spine density in c-Fos(+) neurons of KO mice compared to c-Fos(+) WT (Bonferroni’s *post hoc* test, ^∗^*p* = 0.0446), but no significant differences were observed between c-Fos(–) WT and c-Fos(–) KO groups. **(D)** Spine length was slightly increased in WT c-Fos(+) neurons compared to WT c-Fos(–) neurons [two-way ANOVA c-Fos *F*(1,40) = 7.183, *p* = 0.0106; genotype *F*(1,40) = 1.067, *p* = 0.3079; Bonferroni’s *post hoc* test, ^∗^*p* < 0.05]. **(E)** A significant increase in the percentage of larger spines (>1.0 μm^3^) was seen in c-Fos(+) neurons compared c-Fos(–) in both WT and KO [two-way ANOVA, c-Fos *F*(2,123) = 946.1, *p* < 0.0001; genotype *F*(3,123) = 9.739*e*–005, *p* > 0.9999; Bonferroni’s *post hoc* test, ^∗∗^*p* < 0.01, ^∗^*p* < 0.05).

To examine the effects of ephrin-B1 deletion in adult hippocampal astrocytes on remodeling of dendritic spines following contextual learning, coronal hippocampal sections were collected from WT and KO mice 1 h following contextual recall. We used immunostaining against early immediate *gene c-fos* to identify CA1 neurons that were activated during memory recall (red; Figures 1A,B). Dendritic spines were labeled with DiO (green; Figure 1A) to visualize dendritic spines in both c-Fos(+) and c-Fos(−) neurons (Figures 1A,B).

Spine density was significantly higher in trained KO compared to WT (Supplementary Figure 2A and Supplementary Table 1; *t*-test; *t*_(__43__)_ = 2.414, *p* = 0.0201); however, spine volume and length were not different between trained KO and WT animals (Supplementary Figures 2B,C; spine volume: *t*_(__44__)_ = 1.581, *p* = 0.1210; spine length: *t*_(__42__)_ = 0.920, *p* = 0.3626; *t*-test). Interestingly, in addition to the effect of genotype further analysis showed a significant increase in the spine density on c-Fos(+) neurons compared to c-Fos(−) neurons in KO mice [Figure 1C and Table 1; two-way ANOVA, c-Fos *F*(1,48) = 19.91, *p* < 0.0001; genotype *F*(1,48) = 11.55, *p* = 0.0014; interaction *F*(1,48) = 0.4134, *p* = 0.5233; Bonferroni’s *post hoc* test, ^∗∗^*p* < 0.0066 c-Fos(+) KO vs. c-Fos(−) KO; ^∗^*p* = 0.0422 c-Fos(+) WT vs. c-Fos(−) WT]. We also observed higher spine density in c-Fos(+) neurons of KO mice compared to c-Fos(+) WT (Figure 1C; Bonferroni’s *post hoc* test, ^∗^*p* = 0.0446), but no significant differences were observed between c-Fos(−) WT and c-Fos(−) KO groups. When we analyzed spine volume, c-Fos(+) neurons in both WT and KO mice showed a significant decrease in smaller spines and an increase in larger spines (>1.0 μm^3^) with no effect of genotype [Figure 1E and Table 1; two-way ANOVA c-Fos *F*(2,123) = 946.1, *p* < 0.0001; genotype *F*(3,123) = 9.739e−005, *p* > 0.9999; Bonferroni’s *post hoc* test, ^∗∗^*p* < 0.01, ^∗^*p* < 0.05].

The results suggest that increased number of dendritic spines may underlie enhanced contextual memory in astrocyte-specific ephrin-B1 KO mice. While the increase in spine volume is observed on c-Fos(+) neurons in both WT and KO mice, dendritic spine density remains higher in KO compared to WT mice.

### Excitatory Responses Were Enhanced in CA1 Hippocampal Neurons of Trained Ephrin-B1 KO Compared to Trained WT and Naïve KO Mice

**FIGURE 2:**
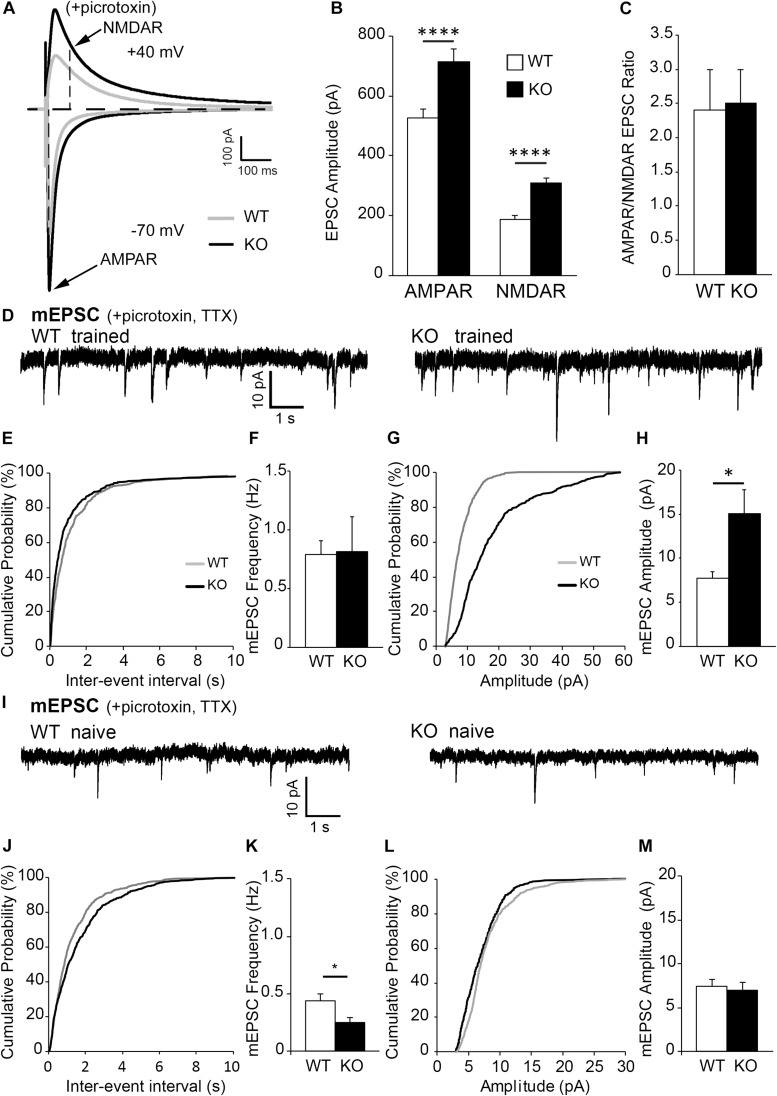
Excitatory post-synaptic responses are enhanced in CA1 hippocampal neurons from astrocytic ephrin-B1 KO mice compared to WT mice. **(A)** Representative traces of excitatory postsynaptic responses in CA1 hippocampal neurons in hippocampal slices from WT (gray) and KO (black) trained mice evoked by stimulating CA3 Schaffer collaterals in the presence of 50 μM picrotoxin, a GABA_A_ receptor antagonist. Neurons were voltage-clamped at either –70 mV to measure AMPAR-mediated EPSCs or +40 mV to measure NMDAR-mediated EPSCs. **(B,C)** Graphs show average EPSC amplitude **(B)** and corresponding ratio of AMPAR- and NMDAR-mediated EPSCs **(C)** (*n* = 12–13 cells, six mice). Evoked AMPAR and NMDAR-mediated currents were significantly increased (AMPAR: *t*_(__398__)_ = 3.568, ^∗∗∗^*p* = 0.0004; NMDAR: *t*_(__373__)_ = 4.61, *p* < 0.0001, *t-*test, ^****^p < 0.0001); however, AMPAR/NMDAR EPSC ratio was unchanged (*t*_(__17__)_ = 0.1495, *p* = 0.8829, *t-*test). **(D)** Sample recordings of mEPSCs from CA1 neurons in hippocampal slices from trained WT and KO mice; recorded in the presence of TTX and picrotoxin (*n* = 6 mice). **(E)** Cumulative probability curve of inter-event intervals between spikes in WT (gray) and KO (black). **(F)** Total average frequency of mEPSCs in WT and KO. **(G)** Cumulative probability curve of mEPSC amplitude in WT and KO. **(H)** Average amplitude of mEPSCs was significantly higher in KO compared to WT (*t*_(__12__)_ = 2.927, ^∗^*p* = 0.0127, *t-*test). **(I)** Sample recordings of mEPSCs from CA1 neurons in hippocampal slices from naïve WT and KO mice; recorded in the presence of TTX and picrotoxin (*n* = 6 mice). **(J)** Cumulative probability curve of inter-event intervals between spikes in naïve WT (gray) and KO (black). **(K)** Total average frequency of mEPSCs in naïve WT and KO. Average frequency of mEPSCs was significantly lower in naïve KO than WT mice (*t*_(__10__)_ = 2.561, ^∗^*p* = 0.0283). **(L)** Cumulative probability curve of mEPSC amplitude in naïve WT and KO. **(M)** Average amplitude of mEPSCs between naïve WT and KO. Error bars represent SEM; ^∗^*p* < 0.05, ^****^p < 0.0001.

Changes in dendritic spine density may affect neuronal functionality; specifically, an increase in dendritic spine numbers in trained KO compared to WT may indicate an increase in excitatory responses. Whole-cell patch clamp experiments were conducted to determine if CA1 hippocampal pyramidal neurons in trained KO mice also show increased excitatory responses compared to trained WT mice. Indeed, increased evoked excitatory responses were observed in CA1 hippocampal neurons of trained KO mice compared to WT mice by measuring both NMDAR and AMPAR currents (Figures 2A,B; WT AMPAR: 527.65 ± 30.30 vs. KO AMPAR: 713.52 ± 43.33, *t*_(__398__)_ = 3.568, *p* = 0.0004, *t-*test; WT NMDAR: 186.36 ± 13.12; KO NMDAR: 307.43 ± 23.59, *t*_(__373__)_ = 4.610 *p* < 0.0001, *t-*test). Interestingly, AMPAR/NMDAR ratio was not significantly different between trained WT and KO mice (Figure 2C; WT: 2.40 ± 0.60; KO: 2.51 ± 0.48, *t*_(__17__)_ = 0.149, *p* = 0.8829, *t-*test). Increased excitatory post-synaptic strength in trained KO mice was further confirmed by increased mEPSC amplitude (Figures 2D,G,H; WT: 7.74 ± 0.73; KO: 15.06 ± 2.76, *t*_(__12__)_ = 2.927, *p* = 0.0127, *t*-test), whereas no differences were observed in mEPSC frequencies between WT and KO trained mice (Figures 2D–F; WT: 0.79 ± 0.12; KO: 0.81 ± 0.30; *t*_(__12__)_ = 0.07389, *p* = 0.9422, *t*-test). In addition, we analyzed mEPSCs in naïve WT and KO mice. We found that mEPSC frequency was reduced in naïve KO compared to naïve WT (Figures 2I–K; WT: 0.44 ± 0.06; KO 0.25 ± 0.044, *t*_(10)_ = 2.561, *p* = 0.0283, *t*-test), but no significant differences in mEPSC amplitude (Figures 2L,M; WT: 7.47 ± 0.75; KO: 7.01 ± 0.92, *t*_(10)_ = 0.3833, *p* = 0.7095, *t*-test). Moreover, two-way ANOVA analysis shows a significant increase of both mEPSC amplitude [two-way ANOVA, training *F*(1,22) = 8.115, *p* = 0.0093; genotype *F*(1,22) = 5.536, *p* = 0.0280, Bonferroni’s *post hoc* test, ^∗∗^*p* < 0.01] and mEPSC frequency in trained KO compared to naïve KO group [two-way ANOVA, training *F*(1,22) = 0.13.99, *p* = 0.0011; genotype *F*(1,22) = 0.4598, *p* = 0.5048, Bonferroni’s *post hoc* test, ^∗^*p* < 0.05]; and supports previously reported biochemical results showing similar increase in synaptic AMPAR levels in trained KO compared to naïve KO group (Koeppen et al., 2018).

It is important to note that inhibitory evoked responses as well as mIPSC amplitude and frequency were not significantly different between WT and KO mice (Supplementary Figure 3), indicating loss of astrocytic ephrin-B1 affects mainly excitatory but not inhibitory function in the adult CA1 hippocampus. Together these results show increased excitability in trained KO mice compared to naïve KO mice, most likely due to increase in the number of excitatory synapses and the recruitment of AMPAR to postsynaptic sites.

### Overexpression of Astrocytic Ephrin-B1 Inhibits New Dendritic Spine Formation on CA1 Neurons Following Learning

**FIGURE 3:**
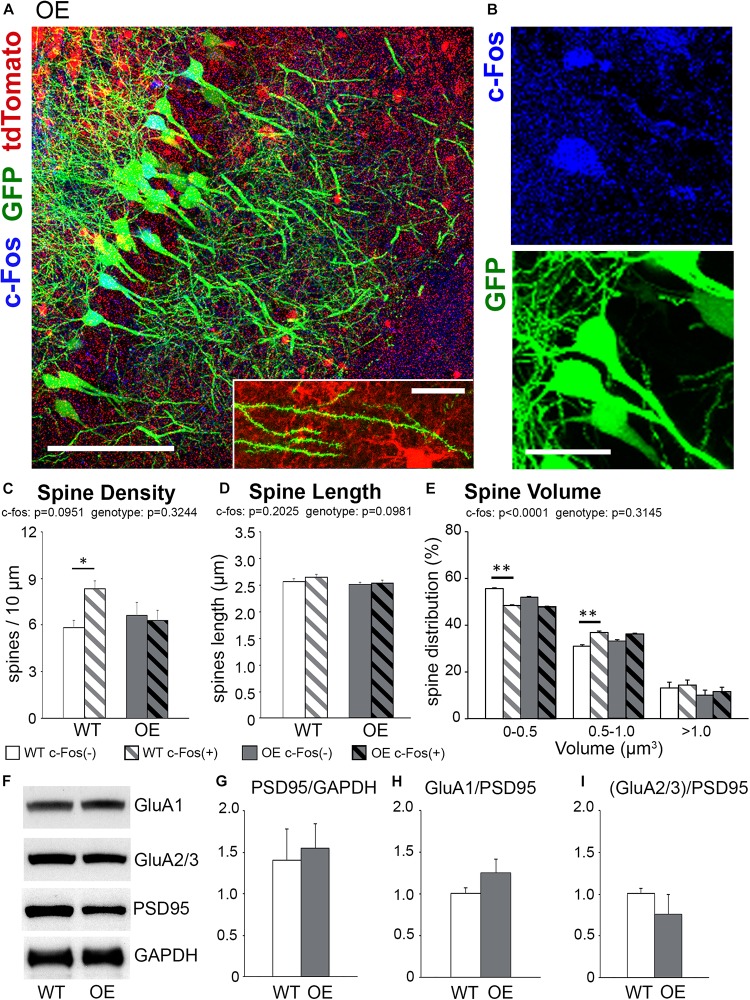
The increase in spine density on c-Fos(+) neurons compared to c-Fos(–) neurons is impaired in OE mice. **(A)** Confocal images of the CA1 neurons expressing GFP (green) and astrocytes expressing tdTomato (red). Some neurons show c-Fos immunoreactivity (blue), scale bar is 100 μm. High magnification image shows example of dendritic spines located in a close proximity to tdTomato-expressing astrocytes, scale bar is 20 μm (insert). **(B)** High magnification images of c-Fos(+) (blue) and GFP-expressing (green) CA1 pyramidal neurons. **(C–E)** Graphs show the average number of dendritic spines per 10 μm dendrite **(C)**, spine length **(D)**, and spine volume **(E)** in c-Fos(+) and c-Fos(–) neurons from WT and OE mice. **(C)** There was an increased dendritic spine density in WT c-Fos(+) neurons compared with WT c-Fos(–) neurons [two-way ANOVA, c-Fos *F*(1,41) = 2.920, *p* = 0.0951; genotype *F*(1,41) = 0.995, *p* = 0.3244; interaction *F*(1,41) = 4.787, *p* = 0.0344; Bonferroni’s *post hoc* ^∗^*p* < 0.05 c-Fos(+) WT vs. c-Fos(–) WT]. **(D)** Spine length was no different between c-Fos(–) and c-Fos(+) neurons in both WT and OE mice. **(E)** A significant decrease in the percentage of smaller spines (<0.5 μm^3^) and an increase in the percentage of larger spines (0.5–1.0 μm^3^) were seen in c-Fos(+) neurons compared c-Fos(–) neurons with no effect of genotype [two-way ANOVA, c-fos *F*(2,141) = 837.4, *p* < 0.0001; genotype *F*(3,141) = 1.194, *p* = 0.3145 Bonferroni’s *post hoc* ^∗∗^*p* < 0.01 c-Fos(–) WT vs. c-Fos(+) WT]. **(F)** Western blots show levels of AMPAR subunits (GluA1 and GluA2/3), PSD95, and GAPDH in synaptosomes isolated from the hippocampus of WT and OE mice 1 h after context A recall. **(G–I)** Graphs show ratios of synaptic PSD95 to GAPDH **(G)**, GluA1 to PSD95 **(H)**, or GluA2/3 to PSD95 **(I)**. Graphs show mean values and error bars represent SEM; ^∗^*p* < 0.05, ^∗∗^*p* < 0.01.

To determine the effects of ephrin-B1 OE in adult hippocampal astrocytes on dendritic spine formation following contextual learning, coronal hippocampal sections were collected 1 h following contextual recall from Thy1-GFP mice containing hippocampal astrocytes expressing tdTomato (WT) or tdTomato with ephrin-B1 (ephrin-B1 OE). We used immunostaining against early immediate *c-fos gene* to identify CA1 neurons that were activated during memory recall [c-Fos(+), blue; Figures 3A,B]. Dendritic spines were visualized with GFP (green, Figure 3A) in both c-Fos(+) and c-Fos(−) neurons and astrocytes expressed td-Tomato (red, Figure 3A).

We observed no significant differences in overall spine density, volume or length between trained OE and WT mice (Supplementary Figures 2D–F; density, *t*-test, *t*_(__42__)_ = 1.463, *p* = 0.1509). However, further analysis showed a significantly higher spine density on c-Fos(+) neurons compared to c-Fos(−) neurons in trained WT but not OE mice [Figure 3C and Table 2; two-way ANOVA, c-Fos *F*(1,41) = 2.920, *p* = 0.0951; genotype *F*(1,41) = 0.995, *p* = 0.3244; interaction *F*(1,41) = 4.787, *p* = 0.0344; Bonferroni’s *post hoc* ^∗^*p* < 0.05 c-Fos(+) WT vs. c-Fos(−) WT]. The impaired increase in spine density on c-Fos(+) neurons compared to c-Fos(−) neurons in OE mice may explain impaired contextual recall in OE mice (Supplementary Figure 1K, *t*-test *p* < 0.05). In addition, a decreased proportion of smaller spines and an increased number of larger spines was seen in c-Fos(+) neurons compared to c-Fos(−) neurons (Figure 3E; two-way ANOVA c-fos *F*(2,141) = 837.4, *p* < 0.0001), but there was no genotype difference [Figure 3E; two-way ANOVA genotype *F*(3,141) = 1.194, *p* = 0.3145]. No significant differences were also seen between trained WT and OE mice in synaptic PSD-95, GluA1 (Figures 3F–H; WT: 1.006 ± 0.063 vs. OE: 1.251 ± 0.161, *t*_(8)_ = 1.637, *p* = 0.140, *t-*test), or GluA2/3 levels (Figures 3F,I; WT: 1.007 ± 0.065 vs. OE: 0.757 ± 0.238, *t*_(8)_ = 1.221, *p* = 0.257, *t-*test).

**TABLE 2:** (Extended data table supporting Figures 3C–E.) Average dendritic spine density, length and volume in Fos(-) and c-Fos(+) CA1 neurons of WT and OE mice.

			Spine distribution (%)		
	Spine sensity (spines/10 μm)	Spine length (μm)	0–0.5 μm^3^	0.5–1.0 μm^3^	>1.0 μm^3^
**WT**					
*c-Fos*(−) (*n* = 10)	5.83 ± 0.47	2.56 ± 0.05	55.65 ± 0.25	31.00 ± 0.52	13.35 ± 0.02
*c-Fos*(+) (*n* = 13)	8.35 ± 0.51*	2.65 ± 0.05	48.58 ± 0.27**	37.03 ± 0.49**	14.43 ± 0.02
**OE**					
*c-Fos*(−) (*n* = 11)	6.60 ± 0.86	2.50 ± 0.05	51.96 ± 0.26	33.36 ± 0.49	10.09 ± 0.93
*c-Fos*(+) (*n* = 11)	6.29 ± 0.67	2.54 ± 0.05	48.05 ± 0.27	36.25 ± 0.46	11.55 ± 0.80

Statistical analysis was performed using two-way ANOVA (genotype and c-Fos as factors) with Bonferroni’s post hoc test: c-Fos(−) versus c-Fos(+): *P < 0.05, **P < 0.01.

Taken together the results suggest that impaired formation of spines on c-Fos(+) CA1 hippocampal neurons may underlie the deficits in contextual recall in astrocyte-specific ephrin-B1 OE mice without affecting dendritic spine maturation.

### Increased Spine Clustering Is Observed on c-Fos(+) Neurons in WT and KO Mice, but Not OE Mice

**FIGURE 4:**
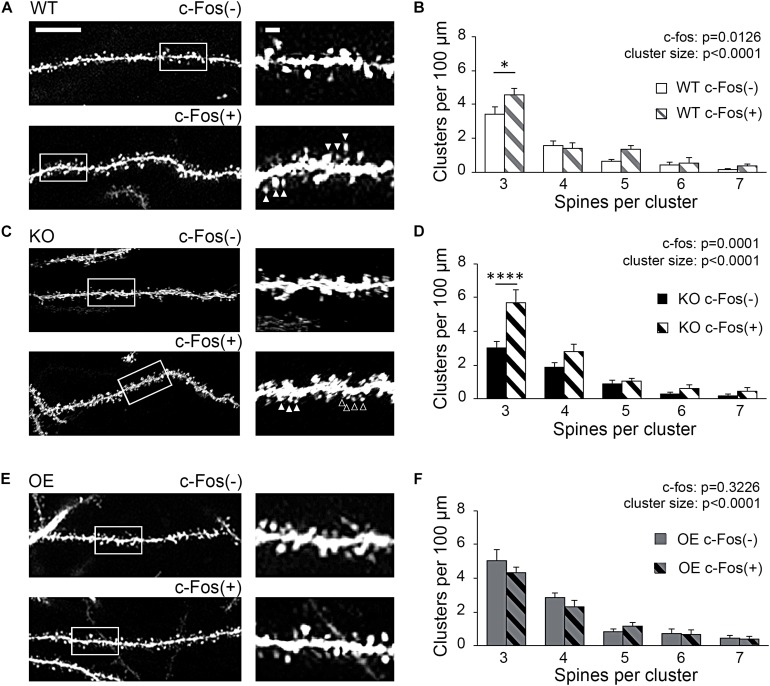
Increased spine clustering is observed on c-Fos(+) neurons in WT and KO mice, but not OE mice. **(A,C,E)** Confocal images of dendritic spines in c-Fos(–) or c-Fos(+) CA1 hippocampal neurons from WT **(A)**, KO **(C)**, and OE **(E)** mice 1 h after contextual recall; scale bar is 10 μm for low magnification images and 2 μm for high magnification images. **(B,D,F)** Graphs show number of clusters containing three, four, five, six, or seven spines (with inter-spine interval < 2 μm) per cluster in c-Fos(–) or c-Fos(+) CA1 neurons from WT **(B)**, KO **(D)** or OE **(F)** mice. **(B)** WT c-Fos(+) neurons had significantly higher number of clusters with three spines than WT c-Fos(–) neurons [cluster size *F*(4,50) = 69.19, *p* < 0.0001; c-Fos *F*(1,50) = 6.698, *p* = 0.0126; two-way ANOVA followed by Bonferroni’s *post hoc*, ^∗^*p* = 0.0109]. **(D)** There was a higher number of clusters with three spines in KO c-Fos(+) neurons compared to KO c-Fos(–) neurons [cluster size *F*(4,130) = 45.77, *p* < 0.0001; c-Fos *F*(1,130) = 15.5, *p* = 0.0001; two-way ANOVA followed by Bonferroni’s *post hoc*, ^****^p < 0.0001]. **(F)** There was no difference in the number of clusters with three spines between OE c-Fos(+) and OE c-Fos(–) neurons. Graphs show mean values and error bars represent SEM; ^∗^*p* < 0.05, ^****^p < 0.0001.

To examine if new spines were added in a close proximity of neighboring spines we analyzed inter-spine intervals (distances between neighboring spines) on c-Fos(+) and c-Fos(−) CA1 neurons in WT mice. As expected, we observed an overall reduction in inter-spine intervals between neighboring spines in c-Fos(+) neurons compared to c-Fos(−) neurons due to an increase in spine density. However, spines were not distributed uniformly as we found a specific increase in the percentage of spines with inter-spine intervals <2.0 μm on c-Fos(+) neurons compared to c-Fos(−) neurons (Supplementary Figures 4A–C; WT c-Fos−: 50.91 ± 1.65 vs. WT c-Fos+: 56.58 ± 1.00, *t*_(__10__)_ = 2.766, *p* = 0.019, *t-*test). We further analyzed clusters of these spines that were <2.0 μm from each other in c-Fos(+) and c-Fos(−) neurons. We observed a significant increase in the number of the spine clusters on c-Fos(+) CA1 neurons compared to c-Fos(−) neurons in WT [Figure 4B; two-way ANOVA; *F*_c–__F__os_(1,50) = 6.698, *p* = 0.0126], in particular smaller clusters containing three spines [Table 3; WT c-Fos− (3): 3.42 ± 0.50 vs. WT c-Fos+ (3): 4.59 ± 0.34; Bonferroni’s *post hoc* test, ^∗^*p* < 0.05]. This suggests that spine formation occurs at specific locations, in a close proximity to neighboring spines, on the dendrites of c-Fos(+) CA1 neurons activated during contextual recall.

**TABLE 3:** (Extended data table supporting Figure 4.) The number of spine clusters with 3, 4, 5, 6, and 7 spines in c-Fos(−) and c-Fos(+) CA1 neurons of WT, KO, and OE mice.

Spine clusters per 100 μm dendritic length					
Spines per cluster	3	4	5	6	7
**WT**					
*c-Fos*(−) (*n* = 6)	3.42 ± 0.46	1.56 ± 0.26	0.62 ± 0.13	0.42 ± 0.14	0.16 ± 0.06
*c-Fos*(+) (*n* = 6)	4.59 ± 0.34	1.41 ± 0.29	1.36 ± 0.21	0.54 ± 0.30	0.38 ± 0.09
Statistics	*t* = 3.232	*t* = 0.404	*t* = 2.032	*t* = 0.333	*t* = 0.593
	*p = 0.0109	*p* > 0.999	*p* = 0.2374	*p* > 0.999	*p* > 0.999
**KO**					
*c-Fos*(−) (*n* = 15)	3.00 ± 0.41	1.84 ± 0.30	0.86 ± 0.26	0.28 ± 0.11	0.18 ± 0.08
*c-Fos*(+) (*n* = 13)	5.67 ± 0.80	2.77 ± 0.48	1.04 ± 0.19	0.61 ± 0.19	0.45 ± 0.18
Statistics	*t* = 5.360	*t* = 1.853	*t* = 0.369	*t* = 0.659	*t* = 0.561
	****p < 0.0001	*p* = 0.3306	*p* > 0.999	*p* > 0.999	*p* > 0.999
**OE**					
*c-Fos*(−) (*n* = 7)	5.03 ± 0.66	2.86 ± 0.27	0.84 ± 0.17	0.70 ± 0.28	0.46 ± 0.16
*c-Fos*(+) (*n* = 7)	4.35 ± 0.29	2.32 ± 0.37	1.18 ± 0.18	0.66 ± 0.31	0.37 ± 0.16
Statistics	*t* = 1.503	*t* = 1.197	*t* = 0.760	*t* = 0.099	*t* = 0.191
	*p* = 0.6906	*p* > 0.999	*p* > 0.999	*p* > 0.999	*p* > 0.999

Statistical analysis of differences between c-Fos(−) and c-Fos(+) expression was performed using two-way ANOVA (c-Fos and cluster size as factors) with Bonferroni post hoc test: *P < 0.05, ****P < 0.001.

Interestingly, we also observed a significant increase in number of spine clusters in c-Fos(+) neurons compared to c-Fos(−) neurons in ephrin-B1 KO mice [Figure 4D, two-way ANOVA *F*_c–Fos_(1,130) = 15.5, *p*_c–Fos_ = 0.0001; Supplementary Figures 4D–F], specifically smaller clusters containing three spines [Table 3; KO c-Fos− (3): 3.00 ± 0.41 vs. KO c-Fos+ (3): 5.67 ± 0.80; Bonferroni’s *post hoc* test, ^****^p < 0.0001]. In contrast, we observed no difference in the number of clusters between c-Fos(+) and c-Fos(−) CA1 neurons in ephrin-B1 OE mice [Figure 4F; two-way ANOVA *F*_c–__F__os_(1,60) = 0.9948, *p*_c–__F__os_ = 0.3226; Supplementary Figures 4G–I].

Astrocytic ephrin-B1 may affect up-regulation of dendritic spine density on c-Fos(+) neurons by impacting new spine formation at selective dendritic domains.

### Synaptic Excitatory Sites Are Up-Regulated in CA1 Hippocampus of Astrocyte-Specific Ephrin-B1 KO Mice Following Fear Conditioning

**FIGURE 5:**
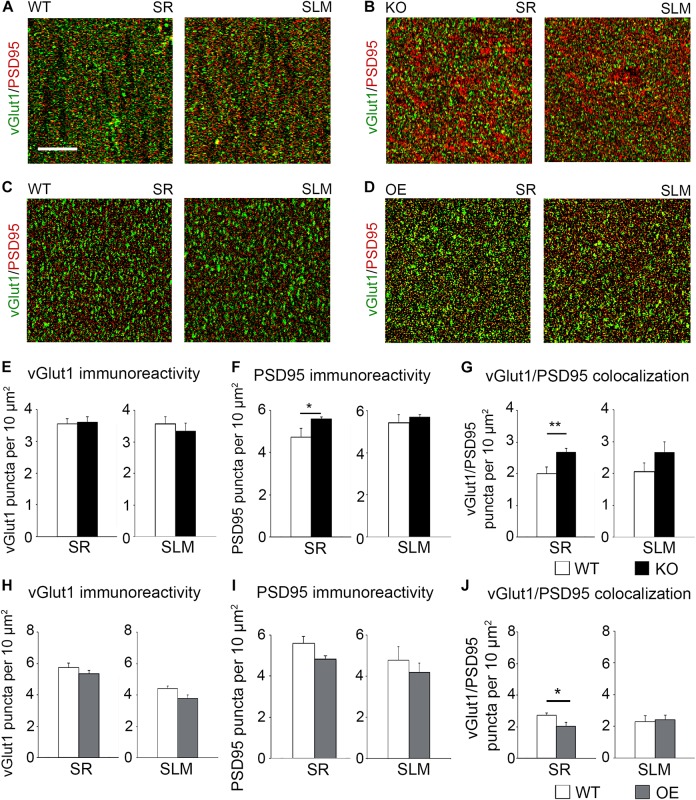
Astrocytic ephrin-B1 negatively regulates the number of excitatory synaptic sites in CA1 hippocampus after fear conditioning. **(A–D)** Confocal images showing vGlut1 (green) and PSD95 (red) immunolabeling in WT **(A,C)**, KO **(B)**, and OE **(D)** in SR and SLM areas of the adult CA1 hippocampus 1 h after contextual recall. Scale bar is 20 μm. **(E–G)** Graphs show the density of vGlut1-positive puncta **(E)**, PSD95-postive puncta **(F)**, and vGlut1/PSD95 co-localization **(G)** per 10 μm^2^ in the SR and SLM areas of the CA1 hippocampus of WT and KO mice. There was no difference in vGlut1 positive puncta between WT and KO mice. However, KO mice showed a significant increase in PSD95 puncta (**F**, *t-*test, *t*_(__32__)_ = 2.104, *p* = 0.043) and vGlut1/PSD95 colocalization (**G**, *t-*test, *t*_(__29__)_ = 2.828, *p* = 0.008) in the SR CA1 hippocampus. Graphs show mean values and error bars represent SEM. **(H–J)** Graphs show the density of vGlut1-positive puncta **(H)**, PSD95-positive puncta **(I)**, and vGlut1/PSD95 co-localization **(J)** in the SR and SLM areas of the CA1 hippocampus of WT and OE mice. There was no significant difference in vGlut1 **(H)** or PSD95 **(I)** puncta between WT and OE mice. OE mice showed a significant decrease in vGlut1/PSD95 colocalization (**J**, *t-*test, *t*_(__32__)_ = 2.433, *p* = 0.022). Graphs show mean values and error bars represent SEM; *p < 0.05, **p < 0.01.

To determine if KO mice also show an increased number of excitatory synapses in the CA1 hippocampus following fear conditioning, excitatory synaptic sites were identified by co-immunostaining against pre-synaptic vGlut1 and postsynaptic PSD95 (Figures 5A,B). Although no changes in vGlut1 positive puncta were detected between trained WT (3.549 ± 0.173) and KO (3.601 ± 0.1753; *t*_(__29__)_ = 0.213, *p* = 0. 833; Figure 5E), a significant increase in vGlut1/PSD95 co-localization was seen in trained KO (2.678 ± 0.116) compared to their WT counterparts (1.999 ± 0.215; *t*_(__29__)_ = 2.828, *p* = 0. 008; Figure 5G). We also observed an increased number of PSD95 positive puncta in trained KO (5.592 ± 0.088) compared to their WT counterparts (4.727 ± 0.425; *t*_(__32__)_ = 2.104, *p* = 0. 043; Figure 5F). In contrast, we observed a significant reduction in vGlut1/PSD95 co-localization (Figure 5G) in trained OE (2.036 ± 0.232) compared to their WT counterparts (Figures 5C,D,J; 2.719 ± 0.158; *t*_(__32__)_ = 2.433, *p* = 0. 022, *t-*test). However, no differences were observed between WT and OE when vGlut1 (WT: 5.736 ± 0.275; OE: 5.352 ± 0.1588, *t*_(__32__)_ = 1.120, *p* = 0.159, *t-*test) and PSD-95 (WT: 5.600 ± 0.336; OE: 4.835 ± 0.148; *t*_(__32__)_ = 2.084, *p* = 0.078, *t-*test) puncta were analyzed separately (Figures 5H,I).

**FIGURE 6:**
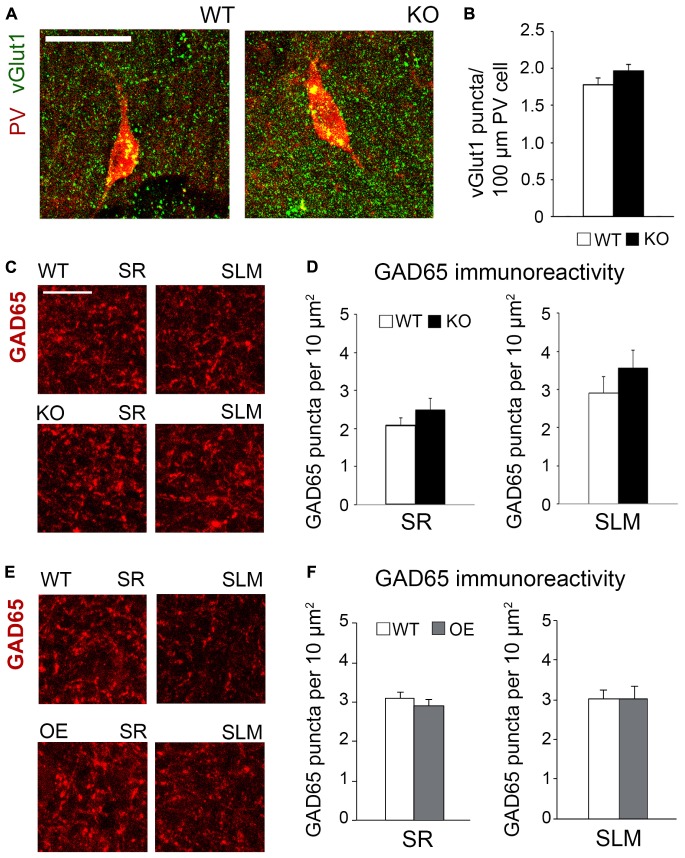
Changes in astrocytic ephrin-B1 levels did not affect the excitatory vGlut1-positive puncta on PV interneurons in SP areas of CA1 hippocampus and inhibitory GAD65-positive puncta. **(A)** Confocal images showing vGlut1 (green) and PV (red) co-immunolabeling in the dorsal CA1 hippocampus of WT and KO adult mice 1 h after contextual recall. Scale bar is 100 μm. **(B)** Graphs show immunoreactivity of vGlut1 positive puncta per 100 μm PV cell in dorsal CA1 hippocampus of trained WT and KO mice. There was no significant difference in vGlut1/PV colocalization between trained WT and KO mice. **(C,E)** Confocal images showing GAD65 (red) immunolabeling in SR and SLM areas of the CA1 hippocampus of KO **(C)** or OE **(E)** mice and their WT counterparts 1 h after contextual recall. Scale bar is 50 μm. **(D)** Graphs show GAD65-positive puncta in the SR and SLM area of the CA1 hippocampus of trained WT and KO mice. There was no significant difference in the number of inhibitory GAD65-positive puncta between WT and KO mice. **(F)** Graphs show GAD65 puncta in the SR and SLM area of the CA1 hippocampus of trained WT and OE mice. No significant differences were seen in GAD65 immunoreactivity between WT and OE mice. Graphs show mean values and error bars represent SEM.

To determine if astrocytic ephrin-B1 also regulates excitatory inputs on inhibitory cells, dorsal hippocampal sections were co-immunostained for vGlut1 and PV (Figure 6A). No significant differences were seen in the number of vGlut1-positive puncta on PV-positive cells between trained WT and KO mice 1 h after contextual recall in SP areas of CA1 hippocampus (Figure 6B; WT: 1.280 ± 0.070 vs. KO: 1.451 ± 0.083; *t*_(__663__)_ = 1.516, *p* = 0.114). We also observed no significant differences in inhibitory GAD65-positive puncta in the CA1 hippocampus between trained WT and KO mice (Figures 6C,D; SR WT: 2.07 ± 0.21; KO: 2.49 ± 0.30; *t*_(__34__)_ = 1.159, *p* = 0.255; SLM WT: 2.90 ± 0.44; KO 3.57 ± 0.46; *t*_(__23__)_ = 1.047, *p* = 0.306) or between trained WT and OE mice (Figures 6E,F; SR WT: 3.10 ± 0.16; OE: 3.02 ± 0.23; *t*_(__31__)_ = 0.9001, *p* = 0.38; SLM WT 3.02 ± 0.23; KO 3.01 ± 0.32; *t*_(__28__)_ ± 0.02563, *p* = 0.98, *t*-test). Whole cell recording from CA1 hippocampal neurons also showed no differences in the amplitude or latency of evoked IPSCs, as well as mIPSC amplitude and frequency between WT and KO mice (Supplementary Figures 3A,B).

The results suggest that excess excitatory synapse formation on excitatory CA1 neurons most likely contribute to enhanced contextual recall in astrocyte-specific ephrin-B1 KO mice, whereas reduced number of excitatory synapses/spines following ephrin-B1 OE in adult astrocytes, in particular on activated c-Fos(+) CA1 neurons, would contribute to impaired contextual recall.

## Discussion

Astrocytes are well positioned to influence learning and memory consolidation by influencing dendritic spine formation and maturation in the adult hippocampus, but molecular mechanisms are not clear. Our data suggest that astrocytic ephrin-B1 controls learning and memory consolidation during contextual fear conditioning by regulating new dendritic spine formation on activated CA1 hippocampal neurons. First, we found that the deletion of ephrin-B1 in astrocytes enhances learning-induced formation of new dendritic spines on CA1 hippocampal neurons, while its OE impairs new synapse formation. Second, ephrin-B1 OE in hippocampal astrocytes selectively affects dendritic spine formation and clustering on hippocampal neurons activated during contextual recall. Third, despite the changes to excitatory synapses, deletion or OE of ephrin-B1 in adult astrocytes does not affect the density of inhibitory GAD65-positive puncta in the CA1 hippocampus. Finally, deletion of ephrin-B1 in astrocytes does not affect learning-induced changes in spine volume, as we observed enlargement of dendritic spines in ephrin-B1 KO mice similar to their WT counterparts. Our results suggest that the deficits in dendritic spine formation and clustering, but not spine maturation, in particular on activated CA1 neurons may underlie impaired contextual memory recall in ephrin-B1 OE mice. These studies implicate astrocytic ephrin-B1 as a negative regulator of synapse formation in the adult hippocampus during learning, which can influence spatial memory.

One major finding of this study is that modulation of ephrin-B1 levels in astrocytes negatively affects the formation of new dendritic spines on activated CA1 hippocampal neurons following learning and contextual recall. Hippocampal excitatory neurons play an integral role in associative memory formation. Activation of CA1 pyramidal neurons is observed during contextual recall in mice (Ji and Maren, 2008). Several studies also report formation of new spines on hippocampal neurons during fear conditioning (Matsuo et al., 2008; Restivo et al., 2009; Giachero et al., 2013; Frank et al., 2018). Indeed, dendritic spines can be considered physical representation of memory (Matsuzaki et al., 2004; Holtmaat and Svoboda, 2009; Kasai et al., 2010). Acquisition of new memories facilitates hippocampal spine formation and spine maturation following contextual fear learning and memory recall, particularly more recent memories (Restivo et al., 2009; Giachero et al., 2013), coinciding with the increased synthesis and recruitment of GluR1 to mature mushroom-type spines in the adult hippocampus (Matsuo et al., 2008). The strong memory trace associated with the fear conditioned response is consistent with an increase of total number of mature dendritic spines. Conversely, extinction of a fear memory induces spine loss, specifically dendritic spines that were formed during the learning phase (Lai et al., 2018). Further, reconditioning following extinction induces formation of new dendritic spines near the sites of spine formation that were induced during initial fear conditioning (Lai et al., 2018). In our study we observed an increase in the number of spines on CA1 neurons in trained astrocytes-specific ephrin-B1 KO mice compared to their WT counterparts, suggesting that astrocytic ephrin-B1 may act as a negative regulator of new spine formation in the adult hippocampus during learning. Astrocytic ephrin-B1 may affect new synapse formation during learning by competing with neuronal ephrin-B for binding to neuronal EphB receptors. Loss of several EphB receptors is known to affect synapse and dendritic spine formation in the hippocampus (Ethell et al., 2001; Henkemeyer et al., 2003).

Another finding of this study is that there is a selective formation of new spines on activated CA1 hippocampal neurons in WT mice. These new spines form in a close proximity of neighboring spines resulting in an overall increase in the number of spine clusters containing three spines. This is consistent with the published work showing that there are hotspots or preferential dendritic regions for spine clustering of two or more spines following contextual fear conditioning (Frank et al., 2018). Clustering of dendritic spines with learning have been demonstrated in layer 5 pyramidal neurons of mouse primary motor cortex following motor learning tasks (Fu et al., 2012) and clusters of axon-dendritic contacts were also observed in vestibular systems of barn owl following prism adaptation (McBride et al., 2008). In our study, we see a selective increase in the number of dendritic spines on activated c-Fos(+) CA1 hippocampal neurons in both WT and KO mice after contextual fear conditioning. However, the increase in spine density is impaired in OE group and we observed no difference in the number of spines and spine clusters between c-Fos(+) and c-Fos(−) CA1 neurons in the presence of ephrin-B1 overexpressing astrocytes. This is potentially due to reduced formation or increased elimination of dendritic spines on CA1 neurons, which most likely underlie impaired contextual recall in OE mice.

While the OE of ephrin-B1 in astrocytes affected spine numbers, the modulation of ephrin-B1 levels in astrocytes did not affect dendritic spine volume. Activity-dependent maturation of hippocampal synapses during memory formation was shown to promote structural changes to dendritic spines (Lichtman and Colman, 2000; Knott et al., 2006; Draft and Lichtman, 2009; Holtmaat and Svoboda, 2009) and to increase synaptic AMPA receptor levels in CA1 hippocampal neurons (Matsuo et al., 2008). Dendritic spines are diverse in structure and undergo activity-dependent morphological changes (Matsuzaki et al., 2004; Matsuo et al., 2008). The structural plasticity of hippocampal dendritic spines allows for spine maturation following learning and memory acquisition (Restivo et al., 2009; Giachero et al., 2013). Neuronal EphB receptors are shown to regulate dendritic spine maturation in hippocampal neurons (Ethell et al., 2001; Henkemeyer et al., 2003) and clustering of AMPARs (Kayser et al., 2006). Activation of EphB2 forward signaling can facilitate the recruitment of AMPARs to synaptic sites (Kayser et al., 2006; Hussain et al., 2015), and ephrin-B reverse signaling can antagonize the internalization of GluR2 subunit of AMPAR allowing for the retention of AMPAR at the cell surface (Essmann et al., 2008). However, our studies show no changes in dendritic spine size between training WT and OE groups. Despite impaired increase in spine density and clustering on the dendrites of c-Fos(+) CA1 hippocampal neurons in OE mice, average size of dendritic spines was not significantly different between WT and KO or WT and OE groups.

Mature spines are larger in size and have larger postsynaptic densities (Harris et al., 1992), allowing for more AMPAR recruitment and anchorage (Ashby et al., 2006; Matsuzaki, 2007). As we observed no differences in dendritic spine size in both KO and OE mice compared to their WT counterparts, we also expected to see normal AMPAR recruitment. Indeed, we detected no differences in synaptic AMPAR levels between the groups, further confirming that the changes in astrocytic ephrin-B1 levels did not affect synaptic AMPAR levels. Although CA1 hippocampal neurons showed increased evoked AMPAR and NMDAR responses in trained KO mice compared to their WT counterparts, the ratio of AMPAR/NMDAR currents was comparable between WT and KO mice suggesting similar mature state of dendritic spines. It is most likely that mESPC amplitude is increased due to an overall increase in the number of functional dendritic spines/synapses on CA1 hippocampal neurons in KO compared to WT mice. In addition, we observed increased mEPSC frequency and amplitude in trained KO mice compared to naïve KO mice, suggesting an increase in number of functional synapses in KO mice following training, which is in agreement with dendritic spine analysis showing an increase in the number of spines on activated c-Fos (+) neurons compared to c-Fos (−) neurons.

Increased AMPAR and NMDAR responses both contribute to enhanced synaptic strength and long-term potentiation (LTP), which is an essential mechanism underlying learning (Bliss and Collingridge, 1993). EphB2 was also shown to modulate synaptic transmission by regulating trafficking and function of NMDAR (Dalva et al., 2000; Henderson et al., 2001; Takasu et al., 2002; Nolt et al., 2011). The ability of synaptic EphB2 receptor to regulate both AMPAR and NMDAR trafficking may influence hippocampal LTP and long-term depression (LTD; Grunwald et al., 2001; Henderson et al., 2001). Indeed, EphB2 loss was shown to attenuate LTP (Grunwald et al., 2001; Henderson et al., 2001) and to impair LTD (Grunwald et al., 2001). While the loss of EphB2 function impairs long-term memory formation, photo-activation of EphB2 using optogenetics during fear conditioning learning enhances long-term memory (Alapin et al., 2018). However, our previous study showed no effects of astrocytic ephrin-B1 deletion on LTP induction and consolidation in the adult hippocampus of naïve WT and KO mice (Koeppen et al., 2018).

Finally, we found no changes in GAD65-immunoreactivity in both ephrin-B1 KO and OE mice. Hippocampal dependent memory formation also requires input from local inhibitory neurons. In fact, ablation of GABA_A_ receptor α5 subunit increased contextual recall (Crestani et al., 2002; Yee et al., 2004) and enhanced spatial learning in mice (Collinson et al., 2002). In addition, an inverse agonist to α5 subunit increased spatial learning (Chambers et al., 2004; Sternfeld et al., 2004). As GABA_A_ receptor α5 subunit is highly expressed on hippocampal pyramidal neurons (Pirker et al., 2000; Rudolph and Mohler, 2006), changes in inhibitory cell activity may be potentially involved in the observed effects of ephrin-B1 KO or OE in astrocytes on memory consolidation. However, after deletion or OE of ephrin-B1 in the adult astrocytes we observed no differences in overall numbers of GAD65 positive sites in the hippocampus of trained mice. Whole cell recording from CA1 hippocampal neurons also showed no differences in the amplitude or latency of evoked IPSCs, as well as mIPSC amplitude and frequency between adult WT and KO mice. In addition, deletion of astrocytic ephrin-B1 did not affect the number of vGlut1-positive puncta on PV-positive inhibitory interneurons in trained KO mice compared to WT mice. Previous studies suggest involvement of hippocampal PV cells in learning and memory. While activation of hippocampal PV interneurons was suggested to contribute to reduced contextual recall after fear extinction (Caliskan et al., 2016), interneurons in CA3 hippocampus expressing high levels of PV were shown to receive higher excitatory input following fear conditioning and also play a role in memory consolidation (Donato et al., 2013, 2015). High-PV expressing interneurons were shown to exhibit a higher excitatory to inhibitory input ratio compared to low-PV expressing interneurons (Donato et al., 2015). Although in our study astrocytic ablation and OE of ephrin-B1 affected the overall number of excitatory sites in the CA1 hippocampus, we did not see changes in inhibitory function between adult KO and WT mice.

**FIGURE 7:**
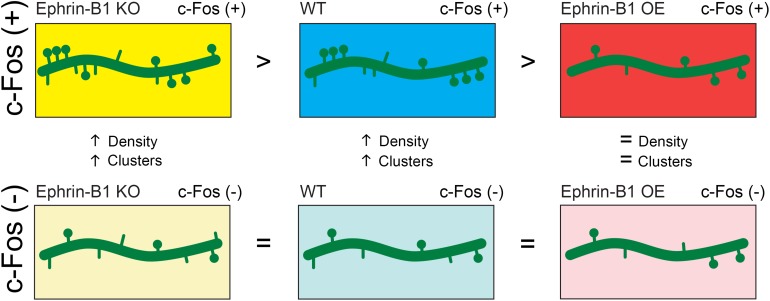
Schematic depiction of the effect of astrocytic ephrin-B1 KO or OE on dendritic spine formation following training. Astrocytic ephrin-B1 regulates excitatory connections in the CA1 hippocampus during contextual memory formation in an activity dependent manner. c-Fos(+) neurons activated during contextual memory recall show higher dendritic spine density and clustering compared to non-activated c-Fos(–) neurons in WT and KO mice. In contrast, no changes in dendritic spine density and clustering were observed between c-Fos(+) and c-Fos(–) neurons in CA1 hippocampus containing astrocytes that overexpress ephrin-B1 (OE). There was a higher number of spines on c-Fos(+) neurons of KO mice compared to WT mice, whereas a lower spine density was observed on c-Fos(+) neurons of OE mice compared to WT mice, coinciding with the enhanced or impaired memory recall, respectively. No differences were detected in spine density on non-activated c-Fos(–) neurons between WT, KO, and OE mice. All together our findings suggest that astrocytic ephrin-B1 is a negative regulator of learning-induced spine formation on activated CA1 neurons.

The studies presented here suggest that astrocytic ephrin-B1 regulates excitatory connections in the CA1 hippocampus during contextual memory formation in an activity dependent manner (Figure 7). While deletion of ephrin-B1 in astrocytes does not affect formation of new spines on activated CA1 neurons, OE of ephrin-B1 in astrocytes impairs it, suggesting that ephrin-B1 is a negative regulator of learning-induced spine formation. Astrocytes have been shown to preferentially contact larger synapses and contribute to synapse stabilization and regulate synaptic activity (Haber et al., 2006; Witcher et al., 2007). However, the role of astrocytes in the formation of new synapses in the adult hippocampus during learning has not been explored yet. We propose that ephrin-B1 plays an important role in astrocyte-mediated new synapse formation during learning. However, it is still unclear whether synaptic activity directly regulate levels of ephrin-B1 in astrocytes and if selective up-regulation or down-regulation of ephrin-B1 in some astrocytes may, respectively, suppress or facilitate new synapse formation at specific dendritic domains induced by local changes in synaptic activity during learning, and potentially underlie memory encoding.

## Data Availability Statement

The raw data supporting the conclusions of this article will be made available by the authors, without undue reservation, to any qualified researcher.

## Ethics Statement

The animal study was reviewed and approved by the UC Riverside Animal Care and Use Program.

## Author Contributions

AN, JK, and IE designed and performed the research, and wrote the manuscript. AN, JK, SW, and KM contributed to the unpublished reagents and analytic tools. AN, JK, SW, KM, ZF, and IE analyzed the data.

## Conflict of Interest

The authors declare that the research was conducted in the absence of any commercial or financial relationships that could be construed as a potential conflict of interest.

## Footnotes

1 http://www.med.upenn.edu/gtp/vectorcore

2 https://imagej.nih.gov/ij/plugins/colocalization.html
