# Supplementary figures and images for: Astrocytic Ephrin-B1 Controls Synapse Formation in the Hippocampus During Learning and Memory
Source: Front Synaptic Neurosci. 2020 Mar 17;12:10. doi: 10.3389/fnsyn.2020.00010 (PMC7092624; doi:10.3389/fnsyn.2020.00010)

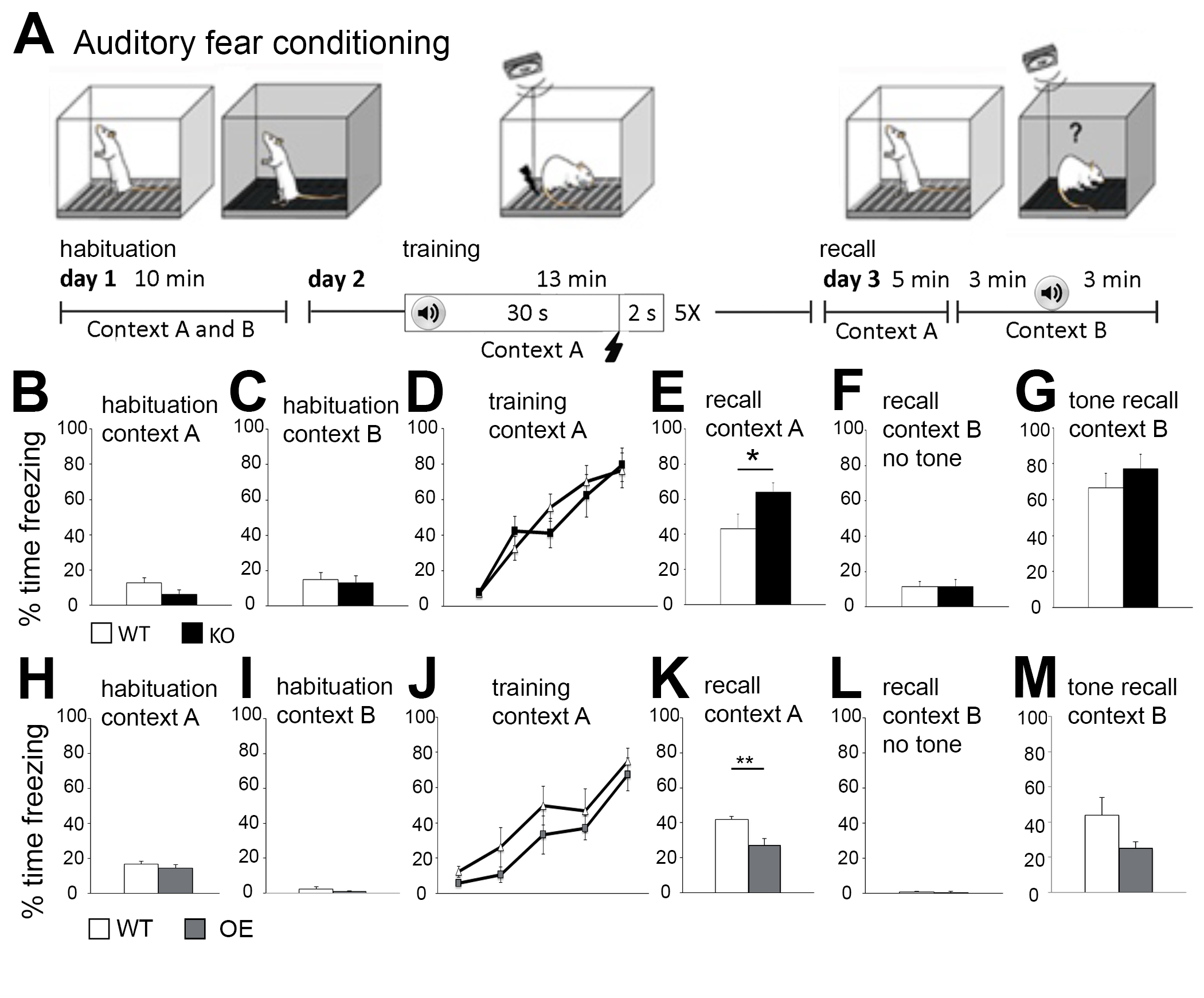

Supplement: Supplementary file 1 [file Image_1.TIF]

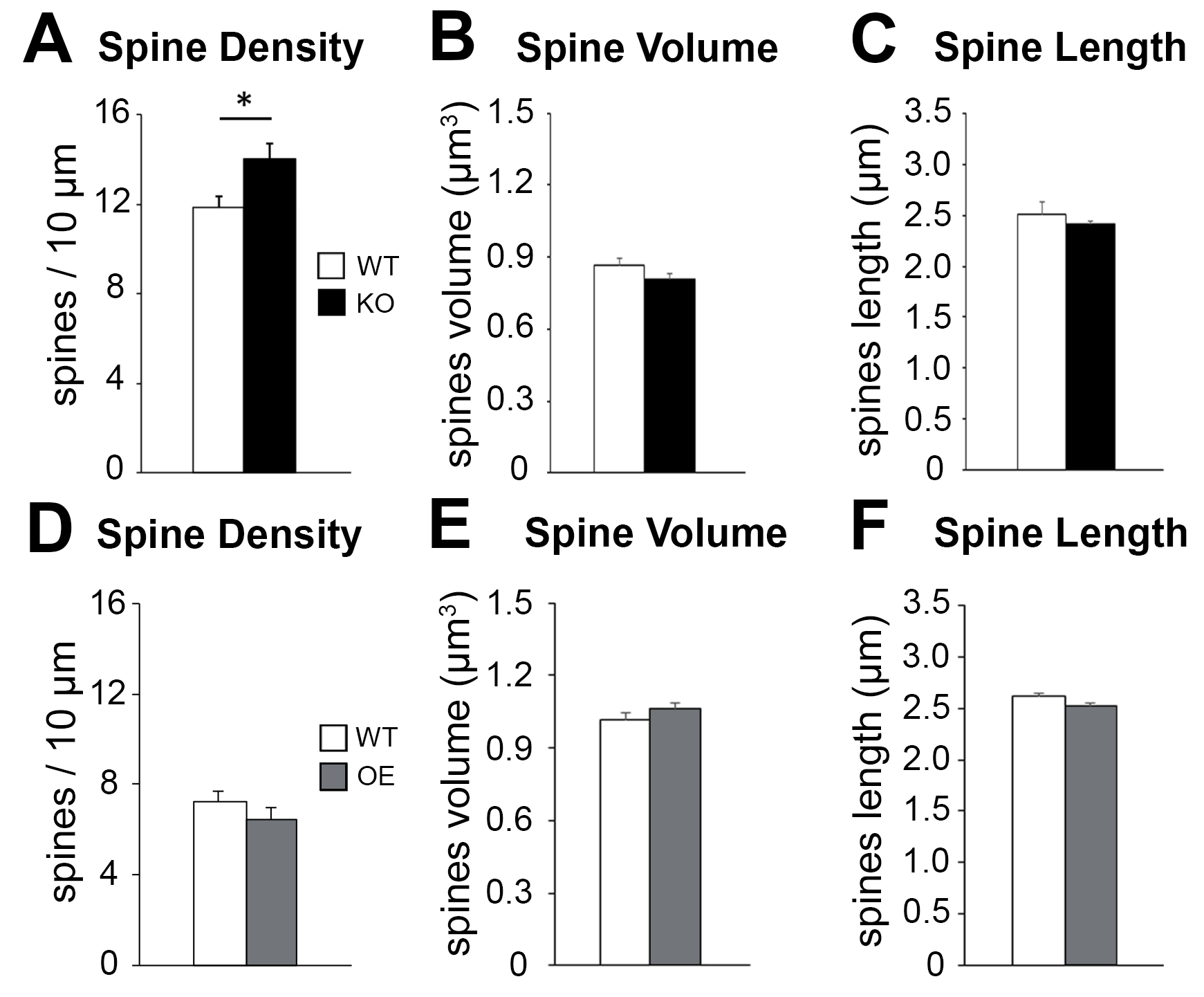

Supplement: Supplementary file 2 [file Image_2.TIF]

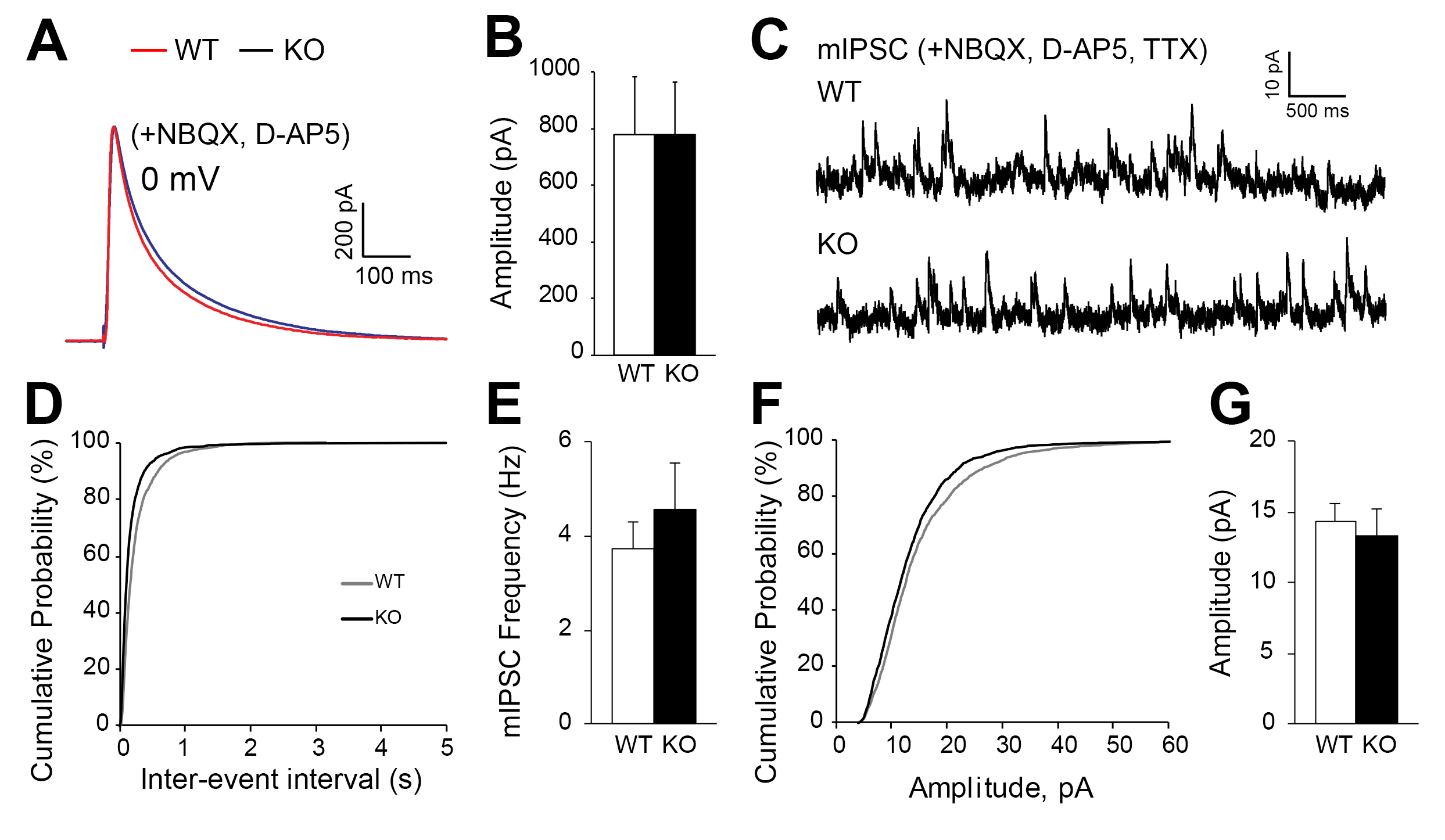

Supplement: Supplementary file 3 [file Image_3.TIF]

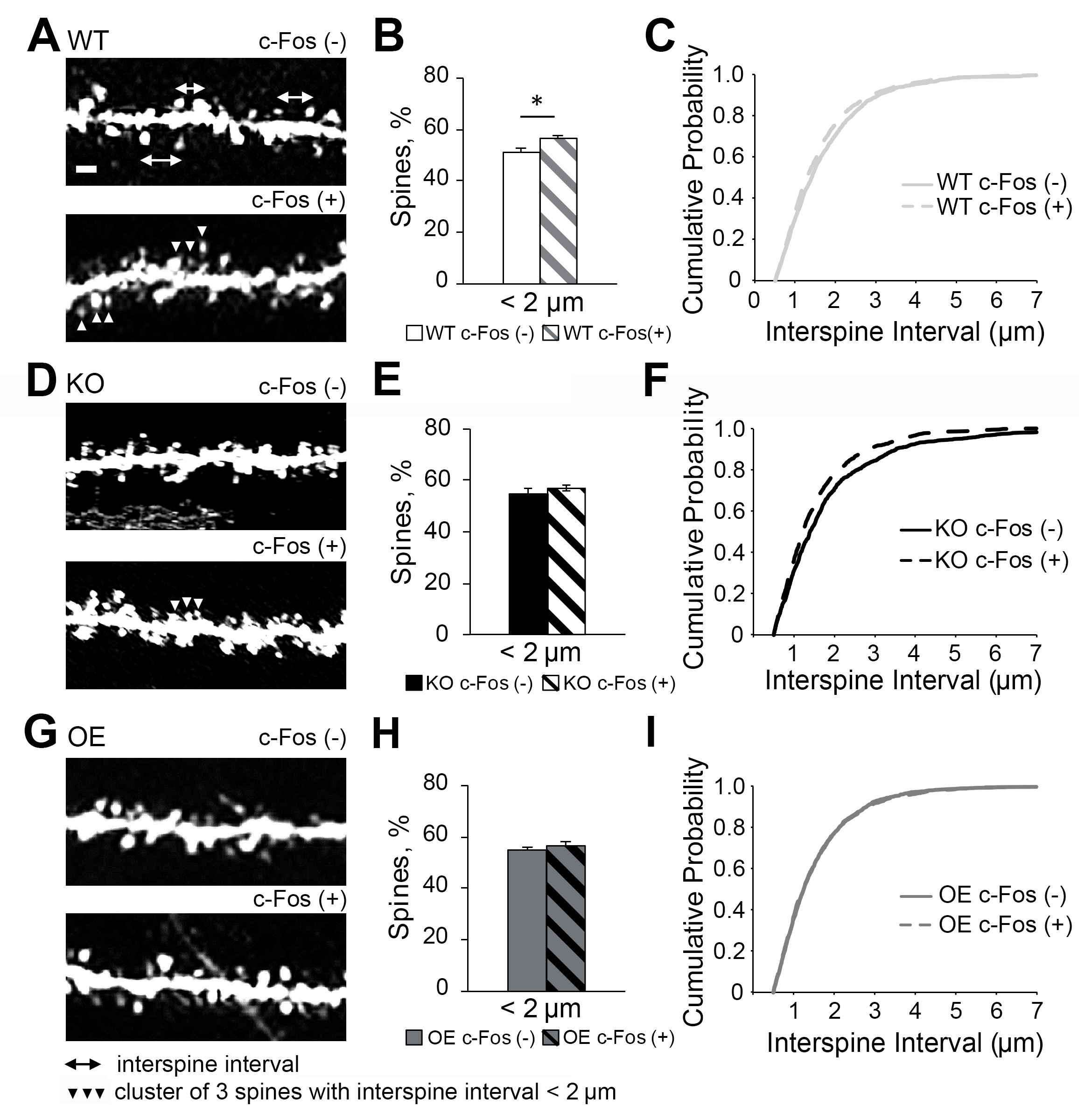

Supplement: Supplementary file 4 [file Image_4.TIF]
