# Supplementary material for: Astrocytic Ephrin-B1 Controls Synapse Formation in the Hippocampus During Learning and Memory
Source: Front Synaptic Neurosci. 2020 Mar 17;12:10. doi: 10.3389/fnsyn.2020.00010 (PMC7092624; doi:10.3389/fnsyn.2020.00010)
Supplement: Supplementary file 5 [file Data_Sheet_1.docx]

**Supplemental Materials**

**Supplemental Table 1** (Extended data table supporting Supplemental Figure 2).

Table shows average dendritic spine density, volume and length in CA1 neurons of trained WT, KO and OE mice. Statistical analysis of differences between trained astrocytic ephrin-B1 KO or OE and its WT counterparts was performed using t-test; *P<0.05.

|  | *Spine Density (spines/10 µm)* | *Spine Volume (µm^3^)* | *Spine Length (µm)* |
| --- | --- | --- | --- |
| **WT** |  |  |  |
| *Trained* (n=21) | 11.85 ± 0.51 | 0.87 ± 0.03 | 2.51 ± 0.12 |
| **KO** |  |  |  |
| *Trained* (n=25) | 14.02 ± 0.68* | 0.81 ± 0.02 | 2.41 ± 0.03 |
|  |  |  |  |
| **WT** |  |  |  |
| *Trained* (n=22) | 7.25 ± 0.44 | 1.02 ± 0.03 | 2.61 ± 0.40 |
| **OE** |  |  |  |
| *Trained* (n=20) | 6.45 ± 0.53 | 1.06 ± 0.03 | 2.52 ± 0.03 |

**Supplemental Figure Legends**

**Supplemental Figure 1** (A) Schematic representation of fear conditioning paradigm. Mice were habituated to contexts A and B for 10 min on day 1 with 1 h gap between context A and context B. On day 2, mice were placed in Context A and received 5 random 0.7 mA foot shocks for 2 s after a 30 s tone of 9 kHz at 70 dB, to train the mice to associate the tone with the foot shock. On day 3 mice were placed in Context A for 5 min, 1 h later mice were placed in Context B for 6 min and exposed to the same tone for the last 3 min. (B-G) Graphs show the percentage of time that KO mice and their corresponding WT mice freeze during each trial, including Context A habituation (B), Context B habituation (C), Context A training (D), Context A recall (E), Context B without tone (F), and with tone (G). KO mice show higher freezing than WT mice during Context A recall (*n =* 7–9 mice per group, t-test; *t*(14) = 2.389 **p* = 0.0315). (H-M) Graphs show the percentage of time that OE mice and their corresponding WT mice freeze during each trial, including Context A habituation (H), Context B habituation (I), Context A training (J), Context A recall (K), Context B without tone (L) and with tone (M). Ephrin-B1 OE mice show reduced freezing compared to WT mice during Context A recall (*n* = 5 mice per group, astrocytic ephrin-B1 OE: 27.27 ± 3.57 vs control: 41.75 ± 2.04, *t*(10) = 3.522, *p* = 0.006, *t* test, ***p* = 0.01). Graphs show mean values and error bars represent SEM.

**Supplemental Figure 2** (A-C) Graphs show dendritic spine density (A), spine volume (B), and spine length (C) in trained WT and KO mice. (A) The total average spine density was significantly higher in trained KO mice than WT mice (*t* test, t_(43)_ = 2.414, p = 0.0201). (B, C) Spine volume and length were similar between WT and KO following fear conditioning (spine volume: t_(44)_ = 1.581, p = 0.1210; spine length: t_(42)_ = 0.9204, p = 0.3626, *t* test). (D-F) Graph shows dendritic spine density (D), spine volume (E), and spine length (F) in trained OE and corresponding WT mice. (D-E) Spine density and volume were similar between trained WT and OE mice (spine density: t_(43)_ = 1.18, p = 0.2447; spine volume: t_(48)_ = 1.036, p = 0.3052, *t* test). (F) Spine length was also not different in OE trained mice (*t* test; t_(47)_ = 1.77, p = 0.0833). Graphs show mean values and error bars represent SEM; *p < 0.05.

**Supplemental Figure 3** (A) Representative traces showing evoked inhibitory postsynaptic currents (IPSCs) recorded in CA1 pyramidal neurons from hippocampal slices of WT (black) and KO (red) mice. (B) Graph shows average amplitude of evoked IPSCs (n=13-14 cells, 6 mice; WT: 778.51 ± 204.61; KO: 779.49 ± 185.15; t_(24)_ = 0.0034, **p* = 0.9973 ; *t* test). (C) Sample recordings of mIPSCs from CA1 neurons in hippocampal slices from WT and KO mice; recorded in the presence of NBQX, D-AP5, and TTX (n = 6 mice). (D) Cumulative probability curve of inter-event intervals between spikes in WT (grey) and KO (black). (F) Total average frequency of mIPSCs in WT and KO. Average frequency of mIPSCs was not significantly different between WT and KO mice (WT: 3.74 ± 0.56; KO: 4.58 ± 0.97, t_(15)_ = 0.6849, **p* = 0.5039, *t* test). (G) Cumulative probability curve of mIPSC amplitude in naïve WT and KO. (H) Average amplitude of mIPSCs between naïve WT and KO. Average amplitude of mIPSC was not significantly different between WT and KO mice (WT: 14.33 ± 1.29; KO: 13.32 ± 1.85, t_(15)_ = 0.4115, **p* = 0.6865, *t* test). Error bars represent SEM.

**Supplemental Figure 4** (A, D, G) Confocal image of CA1 hippocampal dendrites of c-Fos(-) and c-Fos(+) cells in trained WT, KO, and OE mice following fear conditioning; scale bar is 2 µm. White arrows indicate clusters containing 3 or more spines. (B, E, H) Percent of spines located within a distance of 2.0 µm from a neighboring spine in WT, KO, and OE c-Fos(-) and c-Fos(+) mice. WT c-Fos(+) neurons had a significantly higher percent of spines that were located within 2.0 µm distance from neighboring spine (WT c-Fos(-): 50.91 ± 1.65 vs WT c-Fos(+): 56.58 ± 1.00, t(10) = 2.766 **p* = 0.019, *t* test) than WT c-Fos(-) neurons, suggesting an increased spine clustering in WT c-Fos(+) neurons. (C, F, I) Cumulative probability plot of distances between spines in WT, KO, and OE c-Fos(-) and c-Fos(+) mice.
